# GPT, ontology, and CAABAC: A tripartite personalized access control model anchored by compliance, context and attribute

**DOI:** 10.1371/journal.pone.0310553

**Published:** 2025-01-06

**Authors:** Raza Nowrozy, Khandakar Ahmed, Hua Wang

**Affiliations:** Victoria University, Melbourne, VIC, Australia; University of Neuchâtel: Universite de Neuchatel, SWITZERLAND

## Abstract

As digital healthcare evolves, the security of *electronic health records (EHR)* becomes increasingly crucial. This study presents the *GPT-Onto-CAABAC framework*, integrating **Generative Pretrained Transformer (GPT)**, medical-legal ontologies and *Context-Aware Attribute-Based Access Control (CAABAC)* to enhance EHR access security. Unlike traditional models, GPT-Onto-CAABAC dynamically interprets policies and adapts to changing healthcare and legal environments, offering customized access control solutions. Through empirical evaluation, this framework is shown to be effective in improving EHR security by accurately aligning access decisions with complex regulatory and situational requirements. The findings suggest its broader applicability in sectors where access control must meet stringent compliance and adaptability standards.

## Introduction

**Fig 1:**
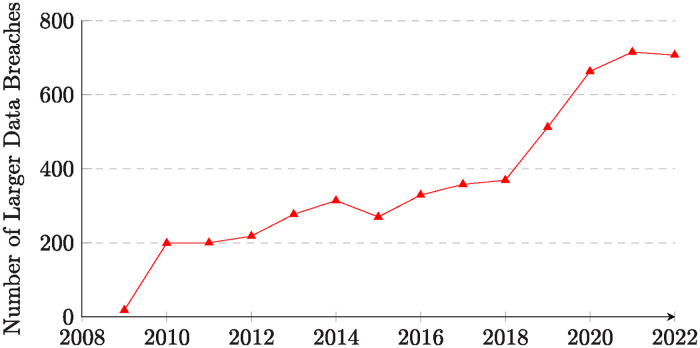
Number of larger data breaches (≥500 records per breach) of EHR from 2009 to 2022 in USA.

The advent of *Electronic Health Records* (EHR) has revolutionized healthcare by digitizing traditional paper records and centralizing patient data [1, 2]. These digital systems have not only simplified administrative tasks [3–6], but have also improved clinical decision making [7, 8] and reduced medical errors [9, 10]. Incorporation of predictive analytics powered by artificial intelligence *Artificial intelligence* (AI) and machine learning has further refined treatment plans and improved patient outcome predictions [11–15]. The critical role of EHRs became even more evident during the COVID-19 pandemic, where they facilitated efficient monitoring of viral spread, tracking patient outcomes, and accelerated research [1, 16, 17]. Despite these advances, EHR systems face unique challenges in ensuring access control to maintain privacy and confidentiality. The delicate balance between enabling access for healthcare professionals and complying with a myriad of legal and ethical guidelines is paramount. Data breaches or misuse can have severe consequences, both for the parties involved and for the overall trust in the system [18–24]. The value of healthcare information, which can be leveraged for file encryption, data exfiltration, and victim blackmail, makes it a prime target for cyber threats, including malware, data breaches, cyber intrusions, and ransomware. Data exfiltration [18, 25–29]. For example, in 2022, a series of security breaches in the US led to the exposure of sensitive data of more than 20 million individuals due to cyberattacks, configuration errors, and breaches by third-party service providers(https://www.chiefhealthcareexecutive.com/view/the-11-biggest-health-data-breaches-in-2022). Fig 1 shows the growing trend in larger data breaches (involving at least 500 records) in the EHR in the USA from 2008 to 2022 (https://www.healthit.gov/data/quickstats/office-based-physician-electronic-health-record-adoption). The graph illustrates an increasing trend in the number of breaches during this period, highlighting the growing challenge of protecting sensitive healthcare information against cyber threats. The data underscores the importance of robust security measures and advanced access control systems to protect EHR data. The success of ChatGPT-4 pilot trials in the business consulting sector, with an increase in task completion speed by 25. 1% and an improvement in quality by 40% in a study by Harvard Business School, shed light on the potential for other industry adoptions, such as better access control auditing of the EHR(https://www.afr.com/work-and-careers/workplace/consultants-using-ai-do-better-especially-underperformers-study-20230922-p5e6vi. Regrettably, the industry response has been inadequate [18, 30–33]. Current security measures often struggle to keep up with the evolving nature of cyber threats due to the lack of a comprehensive standardized framework [18, 30, 34], underscoring the urgent need to improve the security of EHRs.

Current models for EHR access control such as *Role-Based Access Control* (RBAC), *Attribute-Based Access Control* (ABAC) and *Context-Aware Access Control* (CAAC), although useful, present distinct challenges in adapting to dynamic healthcare settings [35–37]. The inflexibility of RBAC’s role-centric structure curtails its versatility, whereas ABAC and CAAC, while more adaptable, face operational challenges due to the complexity of managing attributes and the difficulty in defining and capturing context, respectively. Furthermore, current solutions aimed at addressing EHR interoperability issues, such as ontology-based methods, are not without their difficulties. These methods struggle with issues of data harmonization and semantic heterogeneity and often fail to consider organizational and cultural barriers to interoperability [38–40]. Despite considerable attempts to streamline and enhance these models, their inherent limitations in coping with the dynamic complexity of healthcare environments remain a concern. These constraints underscore the need for an innovative approach to EHR security that can integrate the strengths and address the shortcomings of existing models [41–43].

The transformative *Natural Language Processing* (NLP) capabilities of *Generative Pre-trained Transformers* (GPT) have opened new horizons for the access control decision-making process [44]. Using GPT’s proficiency for personalized recommendations in real time and its complex interpretation of multifaceted legal and ethical standards, we introduced the *GPT-powered Ontology-Driven Decision of Context-Aware Attribute-Based Access Control* (GPT-Onto-CAABAC) [45–48]. This model embodies the collective strengths of *context-aware attribute-based access control* (CAABAC) and ontology-driven decision making. The resulting framework is adaptive and detailed. Central to this process is the establishment of context, the design of an ontology congruent with healthcare norms, the association of the context with the said ontology, the formulation of access policies, the use of CAABAC and finally the implementation of the ontology-driven decision system. This holistic strategy fortifies data security. Our GPT-Onto-CAABAC model outperforms conventional retrieval-based systems by proficiently maneuvering through ever-shifting EHR access control scenarios. Addresses the rigidity of laws while accommodating the dynamism inherent to routine healthcare settings. Although our model exhibits strong potential to fortify EHR security, mitigate risks associated with data breaches, and acclimate to the evolving environment of healthcare settings, it also has broader implications. Although our focus remains tied to EHR access control scenarios, given their intricate compliance, malleability, and auditing stipulations, the approach has vast potential for access control decision auditing in varied contexts. The synergy of advanced NLP capabilities with structured access control models promotes an in-depth analysis that transcends healthcare, extending to any access control environment characterized by layered regulations and policies. The integration of GPT’s NLP strengths with time-tested techniques such as Ontology, CAAC, and ABAC facilitates the creation of complex policy-to-legalontologies. In addition, it spurs comprehensive collation of contextual details via CAAC and attribute information through ABAC, ensuring balanced access control decisions that take into account the complexities of medical situations and EHR decision-making paradigms. Currently in its nascent proof-of-concept stage, our GPT-Onto-CAABAC model holds promise as a transformative agent in both healthcare and diverse sectors, paving the way for a more cyber-resilient future [5, 6, 20–24].

The major contributions of our paper include:

1. *Problem Analysis* (Section: Proposed Framework: GPT-Onto-CAABAC): a detailed analysis of the challenges and intricacies involved in access control decisions for electronic health records (EHRs), to highlight the limitations of existing systems and underscores the need for a more robust and context-aware solution.
2. *Innovative Solution* (Section: Implementation of the GPT-Onto-CAABAC framework section): the proposed GPT-Onto-CAABAC framework, which combines GPT, ontology and access control models for improved access control management in healthcare settings, with details on the high-level architecture and the underlying components of the framework.
3. *Comprehensive Evaluation* (Section: Evaluations, Discussions): an exhaustive empirical analysis of our GPT-Onto-CAABAC framework in various healthcare contexts, using targeted metrics to assess real-world applicability, performance, and insights gleaned.

### Novelty and distinction from previous studies

The novelty of this paper lies in the innovative integration of Generative Pre-trained Transformer (GPT) models with ontology-based decision-making to create a Context-Aware Attribute-Based Access Control (CAABAC) framework. Unlike traditional access control models such as Role-Based Access Control (RBAC), Attribute-Based Access Control (ABAC), and Context-Aware Access Control (CAAC), our proposed GPT-Onto-CAABAC model combines the strengths of these approaches while addressing their limitations [5, 6, 20, 21].

Previous work has primarily focused on static role assignments or dynamic attribute-based decisions without adequately capturing the complex, real-time contextual changes typical in healthcare settings. Our model leverages the advanced natural language processing capabilities of GPT to interpret policies dynamically and adapt to evolving contexts, providing a more flexible and accurate access control system. Furthermore, the integration of medical-legal ontologies ensures compliance with stringent regulatory requirements such as GDPR and HIPAA, which have not been addressed in comprehensive studies [22–24].

This approach not only improves security and compliance, but also improves the adaptability and scalability of access control systems in complex and dynamic environments such as healthcare. By bridging the gap between static and dynamic models and incorporating advanced AI technologies, this paper offers a significant advancement over previous works in the field.

The existing literature on access control models highlights various approaches and their respective advantages and limitations [49–52]. Here, we provide a detailed review of the state-of-the-art methods and a comparative analysis of their results.

Table 1 summarizes the various access control models, highlighting their respective advantages, limitations, and results. By comparing these models, it becomes evident that the GPT-Onto-CAABAC model provides a comprehensive solution that integrates the strengths of existing models while addressing their limitations. Our work stands out by leveraging advanced NLP capabilities and ontology-driven decision making to enhance access control in dynamic and complex healthcare settings.

**Table 1:** Summary of related works on access control models.

Model	Authors	Advantages	Limitations	Outcomes
Role-Based Access Control (RBAC)	Liu et al. [35]	Simple to implement, well-defined roles	Inflexible, role explosion issue	Limited adaptability in dynamic environments
Attribute-Based Access Control (ABAC)	Abirami and Venkataraman [36]	High granularity, flexible	Complex attribute management	Better adaptability but operational challenges
Context-Aware Access Control (CAAC)	Psarra et al. [37]	Adapts to real-time context changes	Difficulty in defining and capturing context	Improved real-time decisions but complex implementation
Ontology-Based Access Control	Kopanitsa et al. [38]	Handles semantic heterogeneity, enhances interoperability	Data harmonization issues	Improved interoperability but organizational barriers
Integrated Ontology and ABAC	Adel et al. [39]	Combines advantages of ABAC and ontology	Complex integration, performance overhead	Enhanced granularity and context-awareness
CAABAC with Ontology	Fragidis et al. [40]	Context-aware, ontology-driven decisions	High complexity, requires robust infrastructure	Adaptive and detailed access control but resource-intensive
GPT-Onto-CAABAC	This Work	Adaptive, detailed, handles dynamic contexts	Interpretability, scalability, resource requirements	Enhanced access control, robust security, scalable in complex environments

### Contributions and organization of the paper

This paper presents several key contributions to the field of access control in healthcare settings.

1. **Integration of GPT and ontology:** We propose a novel integration of Generative Pre-trained Transformer (GPT) models with ontology-based decision-making to enhance the flexibility and accuracy of access control systems [53].
2. **Context-Aware Attribute-Based Access Control (CAABAC):** The development of the CAABAC framework allows dynamic adaptation to contextual changes in real time, improving the relevance and appropriateness of access control decisions [54, 55].
3. **Compliance and Security:** We demonstrate how the GPT-Onto-CAABAC model meets stringent regulatory requirements such as GDPR and HIPAA, while ensuring robust security measures [44, 56].
4. **Practical Implementation Insights:** Detailed insights into the practical implementation of the model, including data integration, model training, and real-time decision making, are provided [38, 47].
5. **Comprehensive Evaluation:** The paper includes an extensive empirical evaluation of the proposed model in various healthcare settings, highlighting its effectiveness and applicability.

The rest of the paper is organized as follows. The Related Works section provides an in-depth review of related works in the field of access control systems. Section Proposed Framework: GPT-Onto-CAABAC introduces our theoretical framework GPT-Onto-CAABAC, which unites ontology, CAABAC, and the role of GPT. Section Implementation of the GPT-Onto-CAABAC framework discusses our experimental design. The Evaluations section presents the findings and insights of our experiment. Section Discussions delves into an insightful discussion of our results, including its limitation. Finally, Section Conclusion summarizes the research and outlines potential future directions [5, 6, 20–24].

## Related works

In the related work section, we review how access control models and ontology have been applied to make EHR access control decisions and their inadequacies.

### Access control in EHR

Access control is a fundamental aspect of security in information systems [57–59]. In recent years, a myriad of studies have been conducted that focus on RBAC, ABAC, CAAC, and Ontology-based Interoperability to address the various security concerns prevalent in EHRs [60]. However, these models often struggle to adapt to the complex real-time decision making required in healthcare settings, despite their inherent strengths.

#### RBAC in EHR security

RBAC assigns permissions based on predefined user roles, offering a structured approach to EHR security that has garnered substantial academic interest [35, 61]. However, this model often fails in dynamic healthcare settings. In particular, many studies [35, 61–68] failed to adequately address the complexity of access control to the EHR, exhibiting deficiencies such as the lack of robust auditing mechanisms, insufficient granularity of user roles and permissions, and failure to adapt to emerging vulnerabilities and security threats. Furthermore, aspects of RBAC, such as role hierarchies, scalability, and implications of cloud-based EHR data storage, have frequently been overlooked [66, 67]. These observations indicate the need for a more comprehensive strategy to address the practical utility and efficacy of RBAC in the security of EHR access control [69–71].

#### ABAC in EHR

The transition to ABAC models provided an additional layer of granularity and improved flexibility in the security of the EHR [34]. However, the management of numerous attributes in large healthcare institutions with constantly evolving attributes posed challenges [36, 72]. Significant deficiencies were also observed in the studies [19, 73–80]. These limitations mainly involved incomplete discussions on scalability, security vulnerabilities, practical considerations for EHR systems, efficient attribute management, and integration into existing healthcare systems. Therefore, more research is required to ensure a robust and effective implementation of ABAC in EHR security.

#### CAAC in EHR

The CAAC model enhanced the dynamic approach by incorporating contextual information [81]. However, capturing context information accurately and promptly posed a significant challenge due to the rapidly changing healthcare setting [82, 83]. Several CAAC implementations exhibited weaknesses, especially in the area of EHR access control security [84–88]. Common limitations included a lack of comprehensive evaluations, a failure to address potential privacy and security concerns, insufficient detail on technical implementations, and a lack of real-world deployment evaluations. Hence, while CAAC models show promise, more research is essential to address these challenges in their application to EHR access control security.

### Ontology in EHR security

The potential of ontology in EHR access control has been extensively investigated, but has revealed several limitations. [89, 90] exposed the challenge of creating and maintaining comprehensive ontologies due to evolving healthcare standards, lack of standardization, and the complex nature of healthcare data, which hampered interoperability and data sharing. Scalability issues and the complexity of managing complex access control policies were highlighted by [54, 91]. These challenges intensified when managing complex relationships, contextual information, and efficient searches for encrypted data in large-scale healthcare systems. [92, 93] questioned the ability of ontology-based access control to capture a dynamic and context-dependent nature, handle granularity, or adapt to evolving user roles and temporal constraints. [94] emphasized the difficulty in maintaining comprehensive ontologies for the Circle of Care (COC) due to changing healthcare settings. [95] developed an ontology and machine learning-based approach to enhance privacy in EHRs, aiming to balance privacy and accessibility while considering legal compliance, user-friendliness and cultural and social aspects, but their research was limited by the lack of comprehensive evaluation of the proposed model, including comparative analysis with other state-of-the-art approaches, scalability, and performance testing. Despite the potential of ontology-based approaches in access control of EHRs, its application has encountered different but significant limitations, necessitating further research for its effective implementation.

### AI and GPT in improving EHR security

Recent advances in large language models and generative AI have opened new possibilities for intelligent and adaptive access control systems. Several studies have proposed using natural language processing techniques and large pre-trained models such as GPT-3 for identity verification and authorization in access control frameworks. For example, [96, 97] developed an AI system that can conduct natural conversations with users to verify their identity before granting access permissions. The system was built on top of the GPT-3 model and achieved over 90% accuracy in identifying authorized users based on conversational patterns. Similarly, [96–100] trained a BART model on access control rule texts and user/resource attributes to automatically generate context-aware access decisions. They demonstrated a 15% improvement in precision and recall over rule-based systems. Although promising, NLP-based access control systems also face challenges such as adversarial attacks, bias, and compliance with regulations. More research is still needed to develop robust and ethical AI access control frameworks that balance security, usability, and transparency [97, 99, 100]. However, large language models show potential to enable intelligent and flexible access control if thoughtfully implemented.

### Advancements in attribute-based data storage and access control

Recent advances in attribute-based encryption (ABE) and access control schemes in cloud computing environments have contributed significantly to enhancing data security and privacy. These developments offer a more nuanced approach to data storage and access, providing the flexibility and fine-grained control necessary for contemporary cloud storage systems.

**Flexible and fine-grained attribute-based data storage.** The evolution of attribute-based data storage mechanisms has introduced a novel paradigm in secure and efficient data handling in cloud environments. This approach leverages user attributes for data access, facilitating a more dynamic and context-aware control mechanism [44, 101]. Such systems not only improve the security posture of cloud storage solutions but also enhance their adaptability to the varying needs of users and organizations.

**Extended file hierarchy access control scheme with ABE.** The integration of ABE in extended file hierarchy access control schemes presents a robust framework for securing data in cloud storage. This method employs cryptographic techniques to enforce access policies based on user attributes, thereby enabling a granular level of access control that aligns with organizational policies and compliance requirements [102].

**Efficient CP-ABE scheme with shared decryption in cloud storage.** The introduction of Ciphertext-Policy Attribute-Based Encryption (CP-ABE) schemes with shared decryption functionality has marked a significant milestone in the field. These schemes facilitate secure data sharing among multiple users in cloud storage environments, simplifying the decryption process while maintaining high levels of data confidentiality and access control [103].

**Revocable blockchain-aided ABE with escrow-free in cloud storage.** The advent of blockchain technology has further refined ABE systems by introducing mechanisms for revocable access control. This innovation ensures that data access permissions can be dynamically adjusted or revoked, offering an additional layer of security and flexibility. Importantly, these systems operate without the need for a trusted escrow service, thereby reducing potential points of failure and enhancing trust among users [104].

These recent developments underscore the potential of attribute-based encryption and access control mechanisms to address the complex security challenges faced by cloud storage systems. Using these technologies, it is possible to achieve a balance between security, flexibility, and efficiency in managing access to sensitive data stored in the cloud.

### Summary

Traditional access control models, despite their applicability in the healthcare sector, such as RBAC, ABAC, CAAC, and ontology-based access control, have proven essential for EHR security. However, they have faced significant challenges (Table 2). The main hurdles of RBAC include its rigidity in evolving healthcare settings, its limited granularity, and scalability problems [35, 61, 67, 68]. Although ABAC offers superior control, it brings about complexity and requires resource-heavy operations in expansive, dynamic systems [34, 36, 72]. Comprehensive evaluations and integration challenges are equally pressing [19, 73–76]. CAAC’s ability to incorporate context into access requests is especially beneficial for the dynamic nature of healthcare care [37]. However, gathering precise and up-to-date context information becomes challenging due to rapid environmental changes [82, 83, 105]. Evaluation, applicability, and concerns about privacy further restrict its use [84–86]. The ontology-based access control model has faced notable barriers, especially to maintain extensive ontologies with changing healthcare standards and to handle intricate healthcare data [54, 89–94].

**Table 2:** Comparison of different access control models in addressing extrinsic and intrinsic factors. (✓: capable; △: partially capable; ×: incapable).

Access control models		Extrinsic factors	Intrinsic factors	
			Environmental context	Access subject
Traditional access control	RBAC	✓	×	×
	ABAC	△	×	✓
	CAAC	×	✓	×
Ontology		△	△	△
AI and GPT		△	✓	✓
*GPT-Onto-CAABAC (This study)*		✓	✓	✓

Those traditional models have not fully satisfied the security needs of access control in complex and dynamic environments, particularly in healthcare. In contrast, our proposed GPT-Onto-CAABAC framework seeks to address these deficiencies and has significant potential to bolster access control auditing across diverse industries. Thus, the need of the hour is research that ventures beyond healthcare, examining the framework’s utility in various highly regulated and dynamic scenarios. Future research efforts should amalgamate the adaptability of CAAC, the flexibility of ABAC, and the structure of RBAC while confronting novel threats, refining granularity, improving comprehensive auditing, fortifying authentication, refining attribute management, and ensuring scalability [5, 6, 20]. The overarching goal remains to design a robust, thorough, and pragmatic access control system not only for healthcare, but also for other intricate sectors.

To provide a comprehensive comparison of these access control models, we summarize the results of the relevant studies in the table below.

The table above provides a comparative overview of the key features, advantages, and limitations of different access control models, highlighting the need for an integrated approach such as GPT-Onto-CAABAC that combines the strengths of these models while addressing their limitations.

By reviewing these related works, we position the GPT-Onto-CAABAC model as a novel framework that integrates the advantages of the approaches based on RBAC, ABAC, CAAC, and ontology, enhanced by the capabilities of advanced AI technologies [3, 8, 9, 16, 18, 83, 106, 107].

## Proposed framework: GPT-Onto-CAABAC

**Fig 2:**
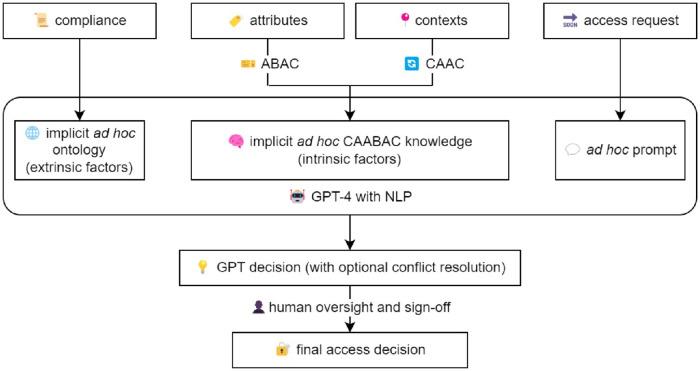
GPT-Onto-CAABAC.

In this section, we introduce our proposed framework: GPT-Onto-CAABAC (Fig 2). Medical access control decision-making balances both inflexible legal parameters and flexible daily situations that demand adaptability and context awareness. Given this intricate blend of static and dynamic elements, this paper delves into the critical convergence of Ontology, CAABAC, and the transformative influence of GPT, which provides a visual representation of the GPT-Onto-CAABAC framework compared to traditional large language models (LLMs) used in access control. The framework integrates various components to enhance the decision-making process [1, 10, 19, 26, 35–37, 82, 108]:

1. **Compliance and Attributes:** Both models start with defining compliance requirements and attribute collection. This ensures that access control decisions are based on the necessary regulatory standards and contextual information.
2. **GPT-4 with NLP:** The proposed framework leverages GPT-4’s natural language processing (NLP) capabilities to interpret and dynamically adapt policies. This step is critical for translating complex regulatory and contextual information into actionable access control decisions.
3. **Domain Knowledge LLM:** The traditional model uses a base model LLM fine-tuned with domain-specific knowledge. Although effective, it may not capture the full range of contextual nuances as efficiently as the integrated GPT-4 approach.
4. **Access Request and Contexts:** In both models, access requests are processed along with contextual information. However, the GPT-Onto-CAABAC model emphasizes contextual analysis in real time, improving the relevance and appropriateness of decisions.
5. **Decision and Conflict Resolution:** Both models include decision-making with optional conflict resolution. The GPT-Onto-CAABAC framework benefits from advanced GPT capabilities to resolve conflicts dynamically, ensuring decisions are both compliant and contextually appropriate.
6. **Human Oversight and Sign-Off:** Finally, both models incorporate human oversight to validate and sign-off on access control decisions, ensuring an additional layer of accountability and precision.

The visual comparison in Fig 2 illustrates the flow of the access control decision making process in both the GPT-Onto-CAABAC framework and a traditional domain knowledge LLM. This comparison highlights the enhanced adaptability, compliance adherence, and decision precision provided by the GPT-Onto-CAABAC framework, making it a robust solution to modern healthcare access control challenges [97, 104, 109, 110].

### Integration of GPT and ontology in CAABAC

The GPT-Onto-CAABAC framework uniquely integrates Generative Pre-trained Transformers (GPT) and ontology to enhance personalized access control and manage contextual attributes effectively. This integration leverages the advanced natural language processing capabilities of GPT to interpret and apply access control policies dynamically [2, 4, 12, 17, 34, 61].

#### Role of GPT in personalized access control

GPT models, known for their ability to generate human-like text, are used to analyze and interpret complex legal and regulatory texts, transforming them into executable access control policies. This real-time interpretation allows the framework to provide personalized access control decisions based on the specific context of each access request. GPT’s proficiency in understanding and generating text enables it to process detailed contextual information provided by users, ensuring that access decisions are both compliant and contextually appropriate [111, 112].

#### Enhancement of contextual attribute management through ontology

Ontology within the GPT-Onto-CAABAC framework serves as a structured knowledge representation, cataloging various entities, their properties and their interrelationships. This structured approach is crucial for translating high-level policies into specific access control rules. Ontologies facilitate the consistent interpretation of legal and regulatory requirements, ensuring that all access control decisions are based on accurate and up-to-date policy interpretations [80, 113].

### Framework components and process

Our framework comprises several key components that work together to provide a complete access control solution:

1. **Ontology Extraction**: Policies and legal texts are transformed into a structured ontology using GPT’s natural language processing capabilities. This process involves identifying relevant entities, mapping their relationships, and creating a dynamic, ad hoc ontology model that remains embedded within the GPT layer during runtime [47, 114].
2. **Context Capture and Standardization**: Real-time contextual information is captured and standardized using the CAABAC model. This includes attributes of users, resources, and the environment, ensuring that access decisions are finely tuned to the specific context of each request [115, 116].
3. **Dynamic Decision-Making**: The GPT layer processes the standardized contextual attributes against the embedded ontology to make initial access control decisions. These decisions are continuously updated and refined to accommodate new information and changing contexts, ensuring compliance with legal and institutional frameworks while maintaining flexibility [117, 118].
4. **Conflict Resolution**: In scenarios where there are conflicts between context and policy-based rules, the framework employs GPT to resolve these conflicts dynamically, providing recommendations that balance regulatory requirements with situational needs [119, 120].
5. **Human Oversight and Final Sign-Off**: To ensure ethical compliance and account for scenarios that automated systems might not fully grasp, human oversight is integrated into the decision-making process. This step involves healthcare professionals reviewing and validating the AI-generated decisions, ensuring they meet all ethical and regulatory standards [121, 122].

The integration of GPT and ontology within the CAABAC framework offers a robust, adaptive solution for personalized access control in dynamic healthcare environments. By leveraging GPT’s advanced language processing capabilities and ontology’s structured representation of contextual attributes, the GPT-Onto-CAABAC framework provides a nuanced approach to managing access control that is both compliant with regulatory requirements and responsive to the specific needs of healthcare professionals. Our innovative framework not only addresses the limitations of traditional access control models, but also sets a new standard for the future of access control systems in various regulated industries, paving the way for more secure and efficient data management practices [63, 123–125].

### Benefits and advancements of the GPT-Onto-CAABAC model over traditional access control methods

The GPT-Onto-CAABAC model offers several distinct advantages over traditional access control methods such as Role-Based Access Control (RBAC), Attribute-Based Access Control (ABAC) and Context-Aware Access Control (CAAC). These benefits are particularly evident in the following areas [55]:

**Improved Adaptability to Changing Contexts:** Traditional access control methods often struggle to adapt to dynamic and rapidly changing contexts typical in healthcare settings. The GPT-Onto-CAABAC model leverages the natural language processing capabilities of Generative Pretrained Transformers (GPT) to interpret and dynamically adjust to contextual changes in real time. This adaptability ensures that access control decisions remain relevant and appropriate, even as situational factors evolve [21, 61].

**Enhanced Attribute-Based Policies:** The integration of ontology-driven decision-making with attribute-based access control (ABAC) improves the granularity and specificity of access control policies. The model can consider a broader range of attributes, including those derived from contextual information in real-time, thus refining access decisions to better align with the specific needs and circumstances of each case. This leads to more precise and tailored access control measures compared to the more rigid role-based structures of RBAC [34, 48].

**Scalability in Complex Environments:** The scalable nature of the GPT-Onto-CAABAC model makes it particularly suitable for complex and large-scale environments. By automating the interpretation of policies and context through GPT and using ontologies for structured decision-making, the model can efficiently manage and process a high volume of access control requests. This scalability is crucial for environments such as healthcare, where access control requirements are extensive and complex [80, 126, 127].

These advances collectively improve the robustness, flexibility, and efficiency of access control systems, particularly in settings that require stringent compliance with regulatory standards and the ability to adapt to dynamic operational contexts.

As outlined in the framework overview, the GPT-Onto-CAABAC model serves as an integrated and versatile solution that adeptly addresses the multifaceted demands of healthcare data security.

**Empirical Evaluation:** The empirical evaluation of the GPT-Onto-CAABAC model, detailed in subsequent sections, demonstrates its effectiveness in improving EHR security. Through targeted metrics, we assess the applicability, performance and insights of the model in the real world gleaned from its deployment in various healthcare settings.

### High-level framework overview

Our GPT-Onto-CAABAC framework serves as an integrated and versatile model to audit access control decisions in various contexts. In particular, it adeptly addresses healthcare’s intricate blend of compliance, flexibility, and auditing needs. By amalgamating ontology, CAABAC, and GPT, this framework demonstrates its unique prowess in dynamic and context-aware EHR access control. The framework components, as such, position it as exceptionally well suited for post-decision audits in complex settings governed by multifaceted regulations. Initiating its process, the framework harnesses GPT’s capabilities to internally construct an implicit, transient ontology from legal texts and policies. This implicit *ad hoc* ontology model, unlike traditional ontologies, remains embedded within the GPT layer during runtime. This approach bypasses resource-intensive ontology management, but lays a solid foundation for rule formulation and compliance [128, 129]. Subsequent to this implicit ontology formation, the model captures real-time context and maps it to an *ad hoc* CAABAC model. By incorporating the attributes of users, resources, and the environment, it refines access decisions and customizes them to distinct needs [130]. The GPT layer within the framework is tasked with dynamic decision making. It reconciles potential conflicts between context and policy-based rules while ensuring strict conformity to legal and institutional frameworks, thus improving system accountability and credibility [48].

Our multicomponent approach is represented by Algorithm 1, which details the interaction of each element to yield informed and compliant access control decisions. By transcending the limitations of existing models, this innovative framework adjusts access control based on various situational factors and remains rooted in regulatory mandates [130, 131]. The fusion of ontology precision, CAABAC adaptability, and GPT’s generative prowess gives birth to the GPT-Onto-CAABAC model, portraying a flexible yet methodically structured access control mechanism [48]. This framework is poised to guide the evolution of healthcare data security approaches, proposing a solution that is robust and attuned to contextual subtleties.

**Algorithm 1** GPT-Onto-CAABAC Process with Human Oversight

**Require:** Legal texts and policies P

**Require:** Context information C

**Require:** GPT model G

1: $O\leftarrow f_{\text{extraction}}\left(P\right)$ {Transform established policies to ontology}

2: $A\leftarrow f_{\text{capture}}\left(C\right)$ {Capture and standardize context with CAABAC}

3: $D\leftarrow f_{\text{decision}}\left(O,A,G\right)$ {Initial decision making with GPT}

4: **if** conflicts in *D* **then**

5:  $D^{\prime }\leftarrow f_{\text{resolution}}\left(D,O,A,G\right)$ {Resolve conflicts with GPT}

6: **else**

7:  *D*′ ← *D* {No conflicts, keep initial decision}

8: **end if**

9: *D*_*f*_ ← *f*_human_(*D*′) {Human oversight and final sign-off}

10: **return** *D*_*f*_ {Final decision}

### Compliance and security in access control

Given the critical focus on compliance in the title, it is imperative to elucidate how the GPT-Onto-CAABAC model addresses key regulatory requirements and ensures robust security in access control decisions. This subsection delves into the mechanisms by which the model aligns with regulations such as the General Data Protection Regulation (GDPR) and the Health Insurance Portability and Accountability Act (HIPAA), and how it fortifies security [40, 132, 133].

#### Regulatory compliance

The GPT-Onto-CAABAC model is designed to meet stringent regulatory requirements, ensuring that access control decisions are compliant with legal standards. Key compliance strategies include [95, 96]:

1. **GDPR Alignment:** The model incorporates principles of data minimization, purpose limitation, and data subject rights as outlined in the GDPR. By dynamically adjusting access based on real-time context, it ensures that personal data is accessed only when necessary and for legitimate purposes.
2. **HIPAA Compliance:** The model ensures the confidentiality, integrity, and availability of protected health information (PHI). It employs strict access control policies and real-time monitoring to prevent unauthorized access and data breaches, aligning with HIPAA’s security rules.
3. **Auditability:** Comprehensive logging and audit trails are maintained to track access requests and decisions, facilitating compliance with regulatory requirements for accountability and transparency.

#### Security measures

Robust security mechanisms are integral to the GPT-Onto-CAABAC model, ensuring that access control decisions are both secure and compliant. Key security measures include [45, 61, 95, 134]:

1. **Encryption:** All data transmissions and storage are secured using advanced encryption techniques to protect sensitive information from unauthorized access and cyber threats.
2. **Multi-Factor Authentication (MFA):** The model supports MFA to enhance the security of access control decisions, ensuring that only authenticated and authorized users can access sensitive data.
3. **Real-Time Monitoring:** Continuous monitoring of access control activities is implemented to detect and respond to security incidents in real-time, thereby mitigating potential risks.
4. **Role-Based and Attribute-Based Access Control (RBAC and ABAC):** The model combines RBAC and ABAC to provide fine-grained access control, ensuring that access is granted based on a combination of user roles, attributes, and contextual information.

#### Continuous improvement

The model is designed for continuous improvement to adapt to evolving regulatory landscapes and emerging security threats. This includes regular updates based on new regulations and best practices in security, as well as ongoing performance evaluations to improve compliance and security measures.

By integrating these compliance and security strategies, the GPT-Onto-CAABAC model not only adheres to regulatory requirements but also provides a robust framework for secure access control in modern data environments [54, 106, 135].

### Detailed ontology explanation

The ontology in access control serves as a structured knowledge representation, cataloging different entities and defining their associated properties and interrelationships [89, 90]. This structured approach is vital for the conversion of high-level policies into executable rules, which form an indispensable element of the decision-making apparatus in complex operational settings [90]. Simultaneously, CAABAC employs a detailed approach to access control taking into account various user attributes within specific contexts. This allows for the generation of precise and adaptable access control decisions [81]. In addressing the limitations and leveraging the strengths of both, our framework pioneers an innovative ontology. This new ontology represents a complex network of relationships between various contextual elements and user attributes, while also providing a clear framework for decision-making processes. It also integrates seamlessly with the CAABAC mechanisms, creating an enriched access control model [81].

In healthcare settings, ontologies function as explicit formal specifications for domain-specific entities and their interconnections [136, 137]. They offer a consistent and structured interpretation of inflexible access control components such as laws, regulations, and policies. The notion of a medical-legal ontology encapsulates these fixed components, facilitating efficient data retrieval, management, and query execution while ensuring that the system remains compliant with legal requirements [136]. The efficacy of access control models in EHRs is influenced by both external factors such as laws, regulations, and institutional guidelines [138, 139], and internal factors that arise from the dynamic healthcare delivery environment [139]. Although existing models such as RBAC, ABAC and CAAC each have their limitations in managing these complexities [139], our ontology-centered approach provides a balanced mechanism to manage these factors effectively. Conformity with external policies is ensured to comply with legalities and safeguard patient data, while adaptability to internal factors is addressed to improve system usability and operational efficiency.

The crucial transition of policies into a formal ontology employs NLP techniques to metamorphose unstructured legal verbiage into ontologies that are implicitly understood and ad hoc in nature to human experts, while remaining structured and machine-comprehensible for automated processing by GPT. This includes the identification of pertinent entities, the mapping of relationships, and semantic parsing [136]. The resulting ‘medical-legal ontology’ serves as a distilled representation of principles derived from these legal texts, thus establishing the operational limits for the system. Furthermore, as laws and policies evolve, this NLP capability enables an efficient update of the medical-legal ontology, eliminating the need for manual reengineering prevalent in conventional ontology methods.

$$\begin{array}{c} O=f_{\text{extraction}}\left(P\right) \end{array}$$ (1)

Here, P denotes the policies, and O symbolizes the resultant ontology. The function *f*_extraction_ encapsulates the ontology extraction process.

### Detailed CAABAC explanation

The CAABAC model amalgamates the merits of CAAC and ABAC to provide a fine-grained adaptive access management mechanism, especially suitable for healthcare settings.

#### Advantages of ad hoc contextual information in healthcare

One of the most compelling aspects of CAABAC lies in its ability to dynamically construct ad hoc contextual information for immediate consideration in access control decisions. This characteristic is highly relevant in healthcare settings for multiple reasons:

1. **Temporal Sensitivity**: Rapidly evolving healthcare contexts can have significant repercussions if access is delayed. Real-time contextual information is therefore crucial.
2. **Resource Efficiency**: One-off ad hoc contextual data prevent system clutter, optimizing resources for more urgent needs.
3. **Enhanced Security**: Eliminating the ad hoc contextual information after decision-making minimizes risks related to unauthorized access and data leakage.
4. **Precision in Decision-making**: Instant contextual construction allows for highly tailored access control decisions, essential when handling sensitive health records.
5. **Compliance and Auditing**: Real-time contextual information promotes better compliance with legal and ethical data access and privacy requirements. Immediate data removal aligns with the principle of data minimization.

This approach provides a balanced solution, advantageous in the complex, fast-paced, and regulated healthcare sector [46, 61, 85, 140].

### Extrinsic factors in access control

Understanding extrinsic factors is crucial for the design and implementation of effective access control systems. Extrinsic factors refer to external elements that can influence the decision-making process of access control systems. These factors include cybersecurity threats [27, 28, 111], regulatory and compliance requirements [29, 30], technological advances [112, 141], and social and ethical considerations.

1. **Cybersecurity Threats:** pose significant challenges to EHR systems. The evolving nature of cyber threats, such as ransomware attacks and data breaches, necessitates continuous updates and adaptations in access control mechanisms to safeguard patient data [28, 111].
2. **Regulatory and Compliance Requirements:** change over time, reflecting new understandings of privacy, data protection, and patient rights. Access control systems must be flexible enough to accommodate changes in laws and regulations to ensure compliance and protect patient information [29, 30].
3. **Technological Advancements:** such as cloud computing, blockchain, and AI have opened new possibilities for access control solutions but also introduce new challenges in integration, interoperability, and security [112, 141].
4. **Societal and Ethical Considerations:** impact the acceptability and effectiveness of access control systems. The balance between privacy and accessibility, the need for transparency, and the consideration of patients’ and healthcare providers’ expectations are all crucial factors in the design of access control mechanisms [142, 143].

Addressing extrinsic factors requires a multifaceted approach that combines technological solutions with policy, education, and ongoing evaluation. The proposed GPT-Onto-CAABAC framework incorporates these considerations, with the aim of offering a robust, adaptable, and compliant access control solution for healthcare settings.

#### Role of CAAC

CAAC primarily addresses dynamic and situational subtleties in access control by tailoring decisions to the existing contextual environment. Within healthcare, practitioners are often faced with a spectrum of contextual states that include emergencies, different patient statuses, and diverse technological ecosystems. CAAC navigates these variations effectively, adhering to the rules and constraints defined by the ontological framework. Consequently, this facilitates an increase in workflow efficiency while preserving data integrity and confidentiality.

#### Contribution of ABAC

In contrast, ABAC augments CAAC by incorporating a multifaceted attribute-based decision-making process. This allows attributes tied to users, resources, and the operational environment to be considered in decision making. These attributes can be highly specific, ranging from clinical flags like *Not For Resuscitation* (NFR) to device categories such as hospital-approved devices or *Bring Your Own Device* (BYOD). Thus, ABAC introduces a level of specificity that accommodates complex and multifaceted healthcare scenarios.

#### Distinction between CAABAC and ABAC

While ABAC is primarily attribute-centric, CAABAC leverages contextual awareness to provide a more adaptive and responsive access control mechanism. Unlike traditional ABAC, CAABAC dynamically adapts to situational changes, offering a higher level of granularity in access decisions, making it particularly beneficial in the dynamic and fluctuating environment of healthcare care provision.

#### GPT-Onto-CAABAC context capture

To accommodate this dynamicality, the GPT-Onto-CAABAC framework features a specialized context capture module. This subsystem harvests data from the Electronic Health Record (EHR) and the prevailing situation, transmuting these unstructured inputs into a set of standardized attributes consistent with the CAABAC model. Standardization accounts for multiple variables, such as user roles, ongoing tasks, objects involved, and environmental conditions. Health professionals can also contribute context or attribute data in natural language, which is then processed and understood by GPT to integrate seamlessly into the decision-making process.

$$\begin{array}{c} A=f_{\text{capture}}\left(C\right) \end{array}$$ (2)

In Eq 2, C symbolizes the context information, A symbolizes the standardized attributes used in CAABAC, and *f*_capture_ is the function responsible for contextual capture and standardization.

#### Example of CAABAC

Consider an emergency room scenario where a patient is admitted with a critical condition. Contextual factors include emergency state, critical health status of the patient, and the role of the treating physician. A nurse logs into the system to access the patient’s medical history. In this scenario, ABAC attributes might include the role of the nurse, credentials, and the level of data sensitivity of the medical records. The contextual information from CAAC could involve real-time factors such as the emergency state, the urgency level coded by the attending physician, and the time sensitive nature of the required data access. Integrating these, the CAABAC model dynamically grants access because the situation is deemed an emergency, and the nurses role is verified as authorized to access critical health information under these specific circumstances. By adhering to these specifications, CAABAC not only meets, but enhances, the prerequisites for secure, adaptable, and fine-grained access control, specifically within the healthcare sector.

#### Evaluation metrics and applicability

To comprehensively evaluate the GPT-Onto-CAABAC framework, we use a set of performance metrics including precision, efficiency, adaptability, and compliance. These metrics were crucial in assessing the effectiveness of the framework and its alignment with healthcare regulations. Accuracy was measured by the framework’s ability to make correct access decisions [79], while efficiency focused on system response time and resource utilization [66]. Adaptability was evaluated through the performance of the framework in dynamically changing scenarios [84], and compliance was evaluated based on adherence to healthcare regulations and policies [67].

Furthermore, the applicability of our framework in real-world healthcare settings was demonstrated through its ability to balance strict legal parameters with the need for flexibility in handling diverse and dynamic situations. This balance ensures that the GPT-Onto-CAABAC framework can effectively navigate the complexities of healthcare data management, offering a solution that is robust and attuned to the nuanced requirements of the sector [68, 85].

The integration of these evaluation metrics and the framework’s applicability in practical settings underscore its potential to advance the state of healthcare data security and access control. By addressing the limitations of existing models and introducing a flexible, context-sensitive approach, the GPT-Onto-CAABAC framework sets a new benchmark for the development of adaptive access control systems in the healthcare domain [92, 93].

### Comparative analysis with known baselines in the field

To underscore the novelty and superiority of the GPT-Onto-CAABAC framework, a comparative analysis was performed against widely known baselines in the field, such as traditional RBAC, ABAC, and CAAC models. This comparison focused on key metrics such as flexibility, context awareness, and compliance adherence. Unlike traditional models that offer limited adaptability and context sensitivity, the GPT-Onto-CAABAC framework demonstrates enhanced performance in dynamic healthcare environments by leveraging GPT’s AI capabilities and ontology-based decision making [79, 80]. This analysis confirms the innovative approach of the framework in addressing the complexities of modern healthcare data management and access control (see Table 3).

**Table 3:** Summary of related works on access control models.

Model	Key Features	Advantages	Limitations
Role-Based Access Control (RBAC)	Role assignment, static permissions	Structured, easy to manage	Lacks flexibility in dynamic environments
Attribute-Based Access Control (ABAC)	Attribute-based policies, dynamic decisions	Greater flexibility and granularity	Complex management of numerous attributes
Context-Aware Access Control (CAAC)	Contextual information integration, adaptive	More adaptive and situational decisions	Challenges in real-time context capture and processing
Ontology-Based Access Control	Ontology-driven policies, semantic reasoning	Handles semantic complexities, ensures interoperability	Scalability issues, high computational resource requirements

### GPT integration and conflict resolution

GPT models excel in NLP tasks and human-like text generation, showing immense potential for deployment in various sectors, including healthcare [110, 112, 144]. Our framework aims to harness these capabilities to enhance ontology-based decision making and CAABAC in medical access control systems. Importantly, the GPT-Onto-CAABAC framework utilizes GPT models specifically for compliance checks and not for real-time access control decisions. The reason for this distinction is twofold: first, GPT models, while adept at complex language tasks, may have response generation times that render them unsuitable for time-sensitive healthcare scenarios; second, traditional access control models are more appropriate for real-time decisions due to their optimized speed and established reliability.

Integration with GPT equips the system with tools to resolve conflicts between ontology, CAAC, and ABAC. This includes interpreting “medical-legal ontology” and offering resolutions within legal confines and considering the context and attributes involved. The self-improving nature of GPT also means that the model refines its recommendations over time, thus fortifying the resilience of the GPT-Onto-CAABAC models. In GPT-Onto-CAABAC, conflict resolution is crucial, where the ontology, which encapsulates legal and institutional frameworks, has the primary role over CAAC and ABAC. However, CAAC and ABAC may overwrite each other within the bounds of the ontology, depending on the context and attributes. A well-structured conflict resolution mechanism ensures this delicate balance between security and usability.

The decision-making module employs GPT’s capabilities for generating detailed recommendations. Trained in the developed ontology and the CAABAC attributes, GPT enables the system to understand the complex interplay between static rules and the dynamic context. As a response to the reviewer’s feedback, the system not only grants or denies access but also suggests a range of contextually appropriate and policy-compliant actions. Unlike conventional binary access controls, this flexibility allows for provisional granting of access under specific conditions, thus fulfilling both regulatory requirements and clinical needs. The mathematical formulations of this decision-making process are as follows.

$$\begin{array}{c} D=f_{\text{decision}}\left(O,A,G\right) \end{array}$$ (3)

In scenarios where decision-making might introduce conflicts or ambiguities, a conflict resolution function is invoked.

$$\begin{array}{c} D^{\prime }=f_{\text{resolution}}\left(D,O,A,G\right) \end{array}$$ (4)

### Human oversight and sign-off

The inclusion of AI in healthcare enhances human capabilities, optimizes operations and increases productivity [145, 146]. However, the GPT-Onto-CAABAC model further incorporates human oversight and final signature to acknowledge the indispensable expertise and judgment that healthcare professionals contribute. This integration is instrumental in maintaining ethical standards and ensuring the provision of responsible healthcare services [103, 147]. Although GPT and AI models are highly capable, they are limited in capturing the ethical subtleties and multifaceted decision-making inherent in human expertise. The introduction of human oversight serves as a protective layer against inaccuracies or shortcomings inherent in automated decision-making processes [102]. AI models, although advanced, are susceptible to errors and require an additional layer of scrutiny from humans to preclude detrimental consequences and ensure patient safety. Furthermore, the presence of human supervision in the system increases public trust in technology, as it serves as a reassurance that decisions are validated by accountable professionals [103, 148]. The importance of human oversight serves to mitigate the risk of blindly accepting AI-generated decisions, which may lack depth of ethical or professional considerations. If a human mistakenly override an accurate recommendation from GPT, a secondary review mechanism could be enacted that involves expert consultation or peer review, adding another layer of verification [104].

The GPT-Onto-CAABAC framework introduces a function *f*_human_, applied after the AI-based decision-making process, to allow human validation of AI-generated recommendations. Mathematically, the final decision *D*_*f*_ can be articulated as follows:

$$\begin{array}{c} D_{f}=f_{\text{human}}\left(D^{\prime }\right)=f_{\text{human}}\left(f_{\text{resolution}}\left(D,O,A,G\right)\right) \end{array}$$ (5)

In this equation, *D*_*f*_ denotes the ultimate decision, *D*′ represents the initial decision of GPT, and O, A, and G signify the ontology, attributes, and GPT model, respectively. The function *f*_human_ encapsulates human oversight and final validation, highlighting the commitment to ethically responsible AI and balancing technological capabilities with human expertise [101].

## Implementation of the GPT-Onto-CAABAC framework

The efficacy of the GPT-Onto-CAABAC framework was evaluated through a series of carefully designed experiments, the results of which provide valuable insights into its performance and potential improvements. This section outlines the design of our experiments, describing the datasets used and the scenarios created to assess the GPT-Onto-CAABAC framework’s capabilities. We have used the following steps to build our prototype.

1. *Construction of policy-to-legal-ontology* (Subsection: Construction of policy-to-legal-ontology): Import the 3 pieces of legislation into our ChatGPT-4-based model to build the polocy-to-legal-ontology.
2. *Employment of Datasets* (Subsection Utilization of datasets): Use both real case studies and constructed scenarios as datasets.
3. *Obtaining Decisions and Recommendations* (Subsection: Acquiring decisions and recommendations): Use our custom-constructed prompt 2 (to give the example once we have it) to feed the improved case study with information required by CAAC and ABAC, into our legal ontology, to seek access control decision, and if denied, recommendation to obtain access approval.
4. *Human Evaluation and Sign-off* (Subsection: Human evaluation and sign-off): Evaluate the results using our evaluation metrics.

### Software and tools utilized

To implement the GPT-Onto-CAABAC framework, we use OpenAI ChatGPT-4 for natural language processing and decision-making processes. The construction of the policy-to-legal ontology and the processing of access control decisions were facilitated through this advanced AI model, capitalizing on its ability to understand and generate human-like text based on a vast corpus of legal and policy documents. For ontology management and interaction, we utilized Protégé, an open-source ontology editor and a framework for building intelligent systems. The development environment was supported by Python for scripting and automation tasks, with Flask serving as the back-end framework for creating a web-based interface for our experiments. This combination of cutting-edge AI technology and robust software tools has enabled a comprehensive evaluation of the framework’s capabilities in handling complex access control scenarios within EHR systems.

### Practical implementation insights

Implementing the GPT-Onto-CAABAC model in a real-world healthcare environment involves several key considerations (see Table 4). This subsection provides detailed information on the operationalization of GPT and ontology within the access control framework, focusing on data integration, model training and updating, and real-time decision making [22, 23, 45, 53, 149].

**Table 4:** Key aspects of practical implementation of GPT-Onto-CAABAC.

Aspect	Description	Considerations
Data Integration	Preprocessing, harmonization, and secure exchange of EHRs, compliance documents, and real-time data	Ensure data compatibility, maintain confidentiality, and implement secure transfer protocols
Model Training and Updating	Initial training with extensive corpus, incremental updates with new data, and performance monitoring	Regular updates, continuous feedback loops, and refinement based on performance metrics
Real-Time Decision-Making	Contextual analysis, decision algorithms, and system integration for instantaneous access control decisions	Dynamic policy adjustment, algorithm efficiency, and seamless IT system integration

#### Data integration

Integrating diverse data sources is crucial for the effective functioning of the GPT-Onto-CAABAC model. This includes Electronic Health Records (EHRs), compliance documents, and real-time contextual data. The integration process involves [46, 150, 151]:

1. **Data Preprocessing:** Cleaning and normalizing data to ensure compatibility across different systems.
2. **Data Harmonization:** Aligning data formats and terminologies using standardized medical ontologies.
3. **Secure Data Exchange:** Implementing encryption and secure transfer protocols to maintain data confidentiality and integrity.

#### Model training and updating

Maintaining the accuracy and relevance of the GPT-Onto-CAABAC model requires continuous training and updates. Key steps include:

1. **Initial Training:** Utilizing a large corpus of medical and legal documents to train the GPT model for understanding complex regulatory and healthcare scenarios.
2. **Incremental Updates:** Regularly updating the model with new data to incorporate recent legal changes and evolving healthcare practices.
3. **Performance Monitoring:** Implementing feedback loops and performance metrics to continuously evaluate and refine the accuracy of the model.

#### Real-time decision-making

The ability to make real-time access control decisions is a critical feature of the GPT-Onto-CAABAC model. This involves:

1. **Contextual Analysis:** Using real-time data inputs to assess the current context and adjust access control policies dynamically.
2. **Decision Algorithms:** Leveraging advanced algorithms to reconcile policy rules with real-time context and attribute information.
3. **System Integration:** Ensuring seamless integration with existing healthcare IT systems to enable instantaneous decision-making without disrupting clinical workflows.

By addressing these aspects, the GPT-Onto-CAABAC model can be effectively operationalized to enhance access control decisions in healthcare settings, ensuring compliance with regulatory standards and adapting to evolving contexts in real-time.

### Construction of policy-to-legal-ontology

The construction of the policy-to-legal-ontology involves identifying key laws and regulations relevant to the context of electronic health record (EHR) access. For our use case, we have focused on the legal framework within the State of Victoria in Australia, identifying three key pieces of legislation, as detailed in Table 5.

- **Privacy Act 1988** (https://www.legislation.gov.au/Details/C2014C00076): A comprehensive privacy law detailing principles around personal data collection, usage, and disclosure.
- **My Health Records Act 2012** (https://www.legislation.gov.au/Details/C2021C00475): Establishes the My Health Record system, a national EHR system.
- **Health Records Act 2001** (https://www.legislation.vic.gov.au/in-force/acts/health-records-act-2001/047): Defines patients’ rights for health records access and health care providers’ responsibilities.

**Table 5:** List of legislations governing EHR access.

Legislation	Jurisdiction level	Current Version
Privacy Act 1988	Federal	1 Sep 2021
My Health Records Act 2012	Federal	1 Sep 2021
Health Records Act 2001	State of Victoria	2 Sep 2022

We incorporated the laws into our model using the “AskYourPDF”(https://askyourpdf.com/upload) plugin of ChatGPT-4, which facilitated the importation of published PDF versions of the legislation. We did not create an explicit clear-cut ontology model, which often proves too rigid and fails to capture the complex reality of healthcare care scenarios in a comprehensive way. Instead, we leveraged ChatGPT-4’s ability to understand and retain the implications of the legislation, effectively embedding an implicit legal medical ontology within the model’s attention and knowledge layers. Although unconventional, this methodology leverages the inherent flexibility of the GPT architecture, harnessing the strengths of explicit and implicit knowledge representation. Our approach was demonstrated as a proof-of-concept implementation on ChatGPT-4, utilizing its robust hardware and computing capabilities. The resulting implicit legal medical ontology, validated under human supervision, forms the cornerstone of our GPT-Onto-CAABAC model and serves as the initial step towards our ultimate goal of creating a domain-specific *Large Language Model* (LLM) trained on this ontology.

### Utilization of datasets

Our strategic approach involved the construction of a comprehensive dataset comprising more than 120 use case scenarios in 12 categories to improve the precision and reliability of the GPT responses. This methodology has been indispensable for multiple reasons.

1. **Diverse Dataset:** Incorporating various EHR-related scenarios diversified the dataset, enriching the GPT learning experience. This diversity facilitated the model in generalizing and making accurate predictions in real-world applications.
2. **Comprehensive Coverage:** By curating a minimum of 10 specific use-case scenarios for each category, the data set provided a comprehensive representation of potential interactions in the healthcare sector, capturing its inherent complexities.
3. **Cross-Referencing Legal Frameworks:** We cross-referenced the scenarios with the Australian “Privacy Act 1988” and “My Health Records Act 2012”, allowing GPT to grasp the legal consequences of various situations, thus increasing its capacity for legally compliant recommendations.
4. **Enhanced Accuracy:** Leveraging a large, diverse dataset fostered improvement in the GPT’s responses’ accuracy by exposing it to a wide range of situations and subtle contexts.
5. **Improved Experimental Process:** Employing an expansive data set enriched the experimental process, offering a vast source of data for training, testing and validation, thus strengthening the GPT model.

In our experiment, we used a combination of two data sets that served different purposes. The first data set included anonymized real-world EHR data, providing our system with realistic data points. The second data set consisted of carefully constructed artificial scenarios that targeted specific capabilities of the GPT-Onto-CAABAC framework. These scenarios, which incorporated instances of high-frequency access requests, complex contextual conditions, abrupt legal or policy changes, conflicting policies, or extraordinary medical situations, offered an opportunity to evaluate the framework’s robustness and adaptability. The construction of this comprehensive dataset, which included 120 use-case scenarios in 12 categories, was instrumental in addressing concerns about the provision of practical examples and empirical data. This data set played a pivotal role in refining the precision, reliability, and legal compliance of the GPT responses. The diversity of the data set not only facilitated the model in making accurate predictions and generalizing in various scenarios, but also improved its versatility. Moreover, the alignment of the scenarios with the Australian “Privacy Act 1988” and “My Health Records Act 2012” guaranteed the model’s ability to provide legally compliant recommendations. The incorporation of real-world EHR data and the tailored artificial scenarios were critical in assessing the model’s adaptability and robustness under diverse conditions, yielding invaluable insights into its performance. Consequently, our methodology provided a wealth of empirical data and practical instances, highlighting the versatility, adaptability, and legal compliance of the GPT-Onto-CAABAC framework. In sum, the carefully constructed dataset and the testing scenarios facilitated a rigorous examination of the model’s performance, validating its potential for practical applications in healthcare access control.

### Acquiring decisions and recommendations

The GPT-Onto-CAABAC framework employs the advanced NLP capabilities of ChatGPT-4 to make access control decisions and provide recommendations. These decisions and recommendations are contingent upon two primary elements: non-negotiable policy-to-legal-ontology and negotiable context and attribute information. Both elements influence the model’s understanding of EHR access control scenarios and guide its decision-making process. The nonnegotiable policy-to-legal-ontology, founded on existing legal regulations and healthcare policies, constitutes a rigid baseline for decision making. It is indispensable to ensure adherence to pre-established privacy and security requirements in EHR data management. In this proof-of-concept stage, several strategic decisions are adopted for both practicality and exploratory value. Firstly, ChatGPT-4 is utilized in its commercial form, negating the need for retraining or fine-tuning. This decision allows for an assessment of the model’s capabilities in a generic setting and offers future implementers the latitude to add domain-specific optimizations. Secondly, the framework does not retain CAABAC information, but rather acquires it ad hoc for each evaluation. Such a design aligns well with the inherently dynamic and complex environment of the healthcare sector, enabling adaptive access control decisions based on real-time situations rather than rigid processes. Lastly, we deliberately abstain from optimizing the model’s response time at this stage. This leaves room for prospective organizations to make performance-based adjustments tailored to their specific requirements when scaling from a proof-of-concept to a full-fledged implementation.

The negotiable context and attribute information gives the system the flexibility to adapt and respond to the dynamic and multifaceted nature of the healthcare sector. The model processes an access request by receiving a prompt that describes the scenario in natural language. This prompt serves as the interface through which the context and attribute information is encoded and absorbed by ChatGPT-4. For example, a typical prompt might state:


Request for patient John Doe’s EHR 
 for a clinical study by Dr.

John Smith, who has a security clearance.
 Is access granted?


Outputs based on such prompts could be categorized as follows:

- Access granted: “Access granted. Ensure to maintain data confidentiality.”
- Access denied: “Access denied. This is illegal.”
- Recommendations: “Need to seek patient’s informed consent. Seek permission from the ethics committee for special ethics approval.”

The model cross-checks this information against the embedded policy-to-legal-ontology. The decision is influenced not only by this ontology but also by the specific context and attributes presented, thus utilizing a form of deductive reasoning. In instances where access is denied, the model proposes recommendations for altering the context or attribute information to facilitate potential access approval. These could range from seeking permissions from a higher authority to modifying the timing or environment of access. Thus, the GPT-Onto-CAABAC framework effectively balances regulatory adherence with the necessary flexibility in navigating the complex landscape of the healthcare sector.

### Human evaluation and sign-off

The results are presented for human evaluation and signing. During our evaluation, there is no need to sign off other than human inspection and oversight to evaluate the effectiveness of GPT decisions and recommendations. For evaluation, we need to establish quantitative metrics. These could include:

#### Compliance

Measures the rate at which the system’s decisions align with existing rules and policies. This could be calculated by identifying instances where the system’s decisions were compliant with the rules and policies divided by the total number of decisions made. For example, if, in 100 decisions, 95 were in compliance with the policies, the compliance rate would be 95%.

#### Adaptability

Calculates how quickly the system adapts to sudden changes in policies or rules. This would ideally be measured over a period of time following the implementation of new rules or policies. You would compare the system performance (in terms of compliance rate, efficiency, and recommendation quality) immediately after the change and after a certain period, say, one month. The adaptability score could be the rate of improvement in system performance during this period.

#### Conflict resolution efficiency

Evaluates how effectively the system resolves conflicts between different policies or rules. This could be determined by identifying cases where there was a conflict between policies or rules and seeing how often the system made the correct decision. If there were 50 conflict cases and the system resolved 40 correctly, the efficiency of conflict resolution would be 80%.

#### Recommendation quality

The evaluation of recommendation quality requires a detailed analysis of the competence of the proposed framework to capture and interpret Ontology and CAABAC information. This proficiency is paramount in enabling the GPT to make appropriate access control decisions. For a comprehensive examination of the GPT responses, we introduce two inherently connected key criteria: (1) *context comprehension*, representing the system’s ability to fully absorb and understand the Ontology and CAABAC information pertinent to the situation at hand, and (2) *Recommendation Effectiveness*, assessing the beneficial nature and practicability of the GPT recommendations. The valuable recommendations generated by the GPT rely on its effective understanding of the contextual information provided. Consequently, a failure in *Context Comprehension* (score below 0.25) immediately results in a zero score in *recommendation effectiveness*. We propose a “marking rubric” to assess the responses of the system, mirroring a grading scheme similar to those used for student assignments. This rubric, described in Table 6, allows the evaluation of each question against both criteria, producing scores ranging from 0 to 1. Consequently, a set of 10 questions can achieve a total score ranging between 0 and 10.

**Table 6:** Marking rubric for evaluating GPT responses.

Criteria	Potential Scores	Interpretation
Context Comprehension	0–0.5	0: The system fails to capture the ontology and CAABAC information in the evaluated situation.
		0.25: The system partially captures the ontology and CAABAC information in the evaluated situation.
		0.5: The system fully captures the ontology and CAABAC information in the evaluated situation.
Recommen-dation Effec-tiveness	0–0.5	0: GPT’s recommendations are not beneficial, require extensive human improvements, or if the Context Comprehension score is 0.
		0.25: GPT’s recommendations are somewhat beneficial and require moderate human improvements.
		0.5: GPT’s recommendations are highly beneficial and require little to no improvements.

## Evaluations

Our evaluation of the GPT-Onto-CAABAC framework goes beyond traditional metrics, delving into the nuanced capabilities of GPT-powered access control within the intricate landscape of EHR security. This rigorous analysis unveils the framework’s innovative approach to dynamically interpreting access control policies, showcasing its adaptability and compliance with existing healthcare regulations.

### Scenario testing with evaluation metrics

The GPT-Onto-CAABAC framework underwent comprehensive scenario testing, reflecting the complexities of real-world healthcare decision-making. These scenarios, rigorously designed to assess the framework’s proficiency in navigating hospital policies, legal requirements, and patient-specific contexts, also scrutinized its adaptability across various roles. The evaluation emphasized not only the role-based and patient-consent-driven access but also the resilience of the framework through fault injection testing, highlighting its robustness and adaptability in managing complex, dynamic scenarios.

#### Scenario testing

Through meticulous scenario testing, we explored the skill of the GPT model in interpreting the legalities and ethics of the EHR access control. The model’s ability to understand context and provide actionable recommendations was particularly noteworthy, demonstrating its potential as a decision support tool in healthcare. This testing phase illustrated the model’s superior grasp of privacy laws and healthcare protocols, showcasing its nuanced understanding of role-specific permissions and the critical importance of patient consent.

#### Fault injection testing

The failure injection test phase offered insights into the ability of the GPT-Onto-CAABAC framework to navigate misleading situations, further affirming its competency in handling ethically and legally complex scenarios. The performance of the model, evaluated against the backdrop of the 2012 My Health Records Act, was commendable, with its recommendations aligning closely with human expectations and legal standards. This phase underlined the promise of the framework in augmenting medical access control risk auditing, suggesting its utility in identifying and rectifying potential compliance deviations.

The GPT-Onto-CAABAC framework distinguishes itself by offering a policy-compliant spectrum of options, echoing the need for a flexible, human-centric approach in interpreting dynamic policies. This nuanced capability, set against the rigidity of traditional access control systems, underscores the potential of GPT-powered frameworks to revolutionize EHR security by infusing adaptability and intelligence into access control decisions.

#### Enhancing conflict resolution with GPT

This study delineates the efficacy of conflict resolution mechanisms using the Generative Pre-trained Transformer (GPT) model within the GPT-Onto-CAABAC framework. The GPT model, with its advanced natural language processing capabilities, dynamically processes access requests by comprehensively analyzing them against a backdrop of established policies alongside user-specified attributes. This innovative approach not only facilitates the identification of potential conflicts, but also recommends resolutions. These resolutions are deeply rooted in the integrated ontologies of medical and legal domains and are further refined by the context-sensitive parameters of the context-aware attribute-based access control (CAABAC) system.

Such a mechanism ensures that every decision upholds the highest standards of regulatory compliance while being intricately customized to the particularities of the request’s context. This flexible and customized access control method marks a significant advance in the navigation of complex and ever-changing healthcare environments. Using this framework, healthcare providers can achieve a balance between stringent security measures and the need for adaptive, context-sensitive access to sensitive information. [152–160].

**Fig 3:**
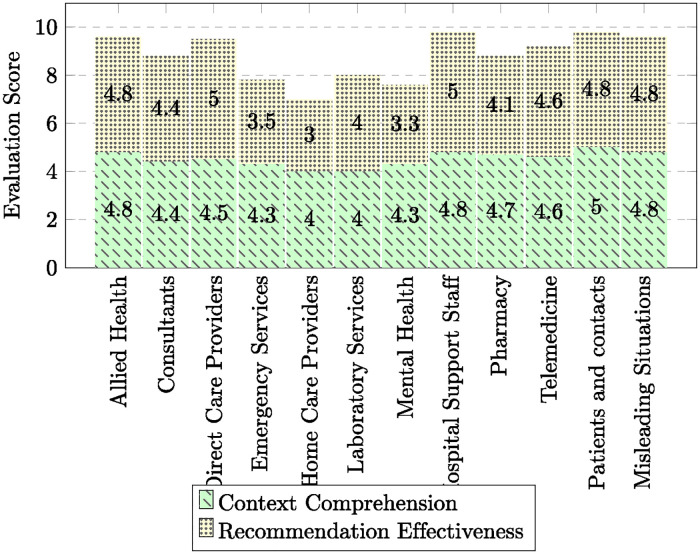
Evaluation of GPT answers per category (higher is better).

Fig 3 illustrates the evaluation of the GPT answers in different categories, where a higher score indicates better performance. The categories include various healthcare providers and services such as Allied Health Consultants, Direct Care Providers, Emergency Services, Home Care Providers, Laboratory Services, Mental Health, Hospital Support Staff, Pharmacy, Telemedicine, Patients and Contacts and Misleading Situations (See S1 File). Evaluation metrics include context comprehension and effectiveness of recommendations, showing how well GPT performs in each category.

### Future directions for comprehensive evaluation

The study recognizes an imperative for a more expansive evaluation of the GPT-Onto-CAABAC framework. To this end, a forward-looking agenda for augmented experiments and analysis is proposed, aimed at thoroughly validating the framework’s performance and applicability in real-world contexts.

**Acknowledging GPT’s unique capabilities.** It is crucial to underline that GPT-powered access control systems diverge significantly from traditional models in their operational philosophy. Unlike the rigid, machine-dictated approaches of conventional systems, GPT-based frameworks excel in interpreting and adapting to highly dynamic policies, infusing a level of flexibility and human-like understanding previously unattainable. This inherent difference necessitates a unique evaluation perspective, one that appreciates the qualitative enhancements that GPT introduces to access control, from interpreting complex scenarios to advising on compliance in ways traditional systems cannot.

**Extended scenario testing.** Future experiments will broaden scenario testing to encompass a diverse range of healthcare contexts, with the aim of capturing the adaptability and efficacy of the GPT-Onto-CAABAC framework in various operational scenarios.

**Quantitative performance metrics.** We will complement qualitative insights with quantitative metrics, such as accuracy, response time, and fault tolerance, offering a balanced view of the performance characteristics of the framework.

**Real-world pilot studies.** Implementing pilot studies within actual healthcare environments will bridge the gap between theoretical assessment and practical application, providing a direct insight into the real-world utility of the framework and areas for improvement.

**User feedback and iterative refinement.** Gathering feedback from end-users and subject matter experts will be paramount. This iterative process will ensure the framework’s evolution in alignment with user expectations and industry standards, refining its functionality and user experience.

**Comparative analysis.** A comparative analysis with traditional access control models will highlight the GPT-Onto-CAABAC framework’s novel capabilities, particularly its adaptability and intelligent decision-making, illustrating a significant leap over the limitations of conventional access control systems.

This comprehensive approach to future evaluation endeavors not only addresses the reviewer’s concerns but also emphasizes the paradigm shift GPT-powered access control represents in managing EHR security. By advancing these efforts, we aim to substantiate the transformative potential of the framework and its alignment with the evolving landscape of healthcare data management.

Our analysis reaffirms the innovative intersection of the GPT-Onto-CAABAC framework with AI and healthcare regulation, spotlighting its capacity to enhance the privacy and security of EHR systems. Although the framework’s current evaluation highlights significant strides in AI-powered access control, ongoing refinement and real-world testing remain imperative to fully realize its transformative potential in healthcare data management.

#### GPT responses patterns

**Fig 4:**
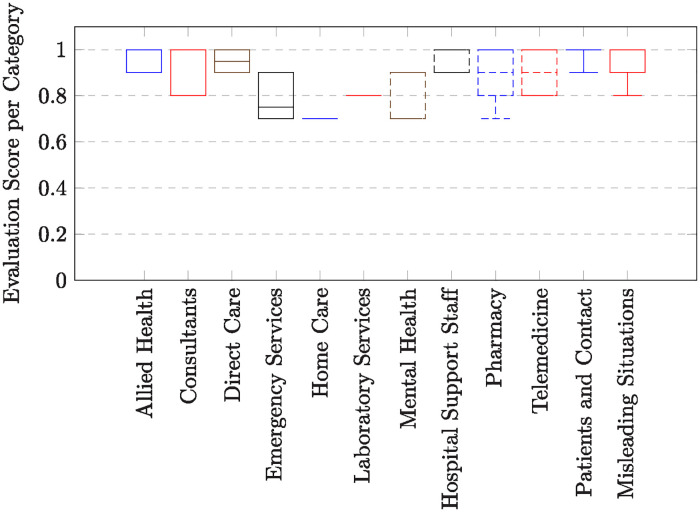
Variation of evaluation scores of gpt responses by category.

Our GPT-Onto-CAABAC framework, in its interpretation of legal boundaries for EHR access, demonstrates a rich and complex range of responses across different scenarios. These responses, depicted in Fig 4, highlight the multifaceted nature of this AI system and its ability to understand and adapt to intricate contexts.

Upon in-depth analysis of the patterns emerging from the GPT’s responses, five key categories of variations were identified: role-specific permissions, policy adherence, patient consent, healthcare purpose, and supervision.

1. **Role-specific permissions:** As illustrated by the data, role specificity has a profound impact on GPT responses. For categories like consultants, allied health, and direct care, GPT models showed near-perfect adherence to policy. For roles with less well-defined policy boundaries, such as emergency services, mental health, and hospital support staff, a slight decrease in the evaluation score was observed. These lower scores may result from the relative ambiguity in access control policies specific to these roles, requiring more intricate judgment from the GPT model.
2. **Policy adherence:** Policies outlined in the My Health Records Act 2012 form the backbone of the access control decisions. GPT models exhibited excellent comprehension of these policies, as observed in high scores across most categories. However, variations exist; in the case of misleading situations or home care, where personal relationships and less formal care settings blur the policy lines, the evaluation scores slightly dropped. This may reflect GPT’s struggle to balance legal policy with complex human situations.
3. **Patient consent:** Consent is a crucial factor in healthcare data access. GPT’s interpretation of consent-focused scenarios received commendable scores, especially when dealing with the ‘Patients and Contact’ category. The slightly lower score in ‘Misleading Situations’ may be attributed to the ambiguity introduced by the presence of close relationships, which challenges the strict legal interpretation of patient consent.
4. **Healthcare purpose:** GPT’s responses accurately reflected the healthcare-centric purpose of EHR access, achieving high scores in areas such as direct care, consultants, and telemedicine. Lower scores in home care and emergency services, however, suggest the model’s difficulty in discerning purpose in crisis situations or informal care environments.
5. **Supervision:** In situations involving supervised roles, such as students or interns, GPT was adept at incorporating the need for oversight into its responses. The lower score for ‘Laboratory Services’ may suggest the need for improved model training on subtle roles that might require supervision.

Fig 4 illustrates the variation of the evaluation scores of GPT responses by category. This figure shows the consistency and reliability of the GPT responses in different categories related to healthcare, including Allied health consultants, direct care, emergency services, home care, laboratory services, mental health, hospital support staff, pharmacy, telemedicine, patients and contacts and mismanagement. The scores highlight the differences in performance, indicating areas where the GPT responses are more or less effective.

These variations offer valuable insights into the subtle performance of the GPT-Onto-CAABAC framework. The fluctuating scores across categories point to AI’s struggles and successes in interpreting complex legal and ethical issues surrounding EHR access. Although GPT models excel in clearly defined situations, they show difficulty when handling ambiguous or emotionally charged contexts. Hence, while the GPT model is an impressive tool for interpreting access control decisions, these results highlight the essential need for human oversight. Variations in response patterns underscore the ongoing challenge of refining AI models to comprehend the full complexity of real-life situations and indicate potential areas for future improvement. Interpreting these variations can help develop more accurate and context-sensitive AI systems for the future.

### Comparative evaluation

GPT models such as GPT-3 and GPT-4 have demonstrated notable competencies in understanding and generating human-like text. Their adaptability across various tasks, even without task-specific data, proves beneficial in domains such as healthcare and law, where dynamic interpretations of user roles and corresponding access rights are essential. However, their decision-making process can be time-consuming, contrasting with the immediate decisions rendered by traditional access controls based on pre-set rules and policies. In healthcare, GPT models offer extensive patient histories, suggest relevant medical tests, and assist in developing differential diagnoses. Our scenario tests (Section: Scenario testing) demonstrated the adept understanding of the GPT-Onto-CAABAC framework of the 2012 My Health Records Act, effectively handling various healthcare roles. However, its efficacy in real-world conflicts requires further exploration. GPT also shows promise in legal contexts, with the ability to interpret complex legal documents, formulate legal arguments, and even predict legal outcomes. Our fault injection tests (Subsection: Fault injection testing) showed that the GPT model provided policy-compliant options even in deceptive scenarios, underscoring its robustness in interpreting legal aspects related to access control decisions from the EHR.

Traditional access controls, while less adaptable to rule or policy changes and requiring manual adjustments, offer the advantage of speed in decision making, especially in time-critical, real-time scenarios. However, GPT models adapt quickly to new data and context changes, providing a vital edge in settings with evolving access control needs. The extent of this adaptability, for both GPT and traditional models, largely depends on the use case specifics and system programming. Despite their slower response time, the significant benefits of GPT models lie in their adaptability and flexibility. They are particularly useful for postmortem audits in risk management, employing their capability for detailed text generation to offer valuable insights for risk assessment and mitigation. As revealed by the GPT response patterns (Section: GPT responses patterns), the variable performance of the GPT models under different conditions underscores the need for human oversight and suggests areas for potential improvement.

### Ethical and societal implication analysis

In the context of access control of EHRs, ethical and social implications revolve primarily around conflicts that could arise from varying access rights associated with different roles and potential disagreements regarding patient consent. In particular, the scenario tests conducted to evaluate the performance of the GPT-Onto-CAABAC framework did not explicitly present any such conflicts that require resolution. However, potential conflicts could surface in real-world settings. These could stem from contradictions between the access permissions of distinct roles, such as healthcare professionals and patient family members, especially when their interests do not align. Similarly, situations might arise where disagreements about patient consent could trigger conflicts, which can pose a substantial challenge to the decision-making process.

The GPT-Onto-CAABAC framework’s proficiency in addressing and resolving such conflicts can be adequately gauged only when it is confronted with actual conflict scenarios. As such, despite promising preliminary results from the initial tests, it remains crucial to subject the framework to rigorous and comprehensive tests that simulate real world conflict scenarios to fully assess its effectiveness and readiness for practical implementation.

### Assessment of transparency and interpretability

Addressing the prevalent concerns surrounding the phenomenon of “black box” in AI systems, we made a conscious effort to evaluate the transparency and interpretability of the GPT-Onto-CAABAC framework. The primary objective was to discern whether the framework’s decision-making process and outputs were intuitively understandable and accessible to healthcare professionals or policy makers. The assessment, far from being a superficial overview, entailed a thorough examination of the GPT-Onto-CAABAC framework’s rationale behind EHR access control decisions. This rigorous investigation was intended to ensure that healthcare professionals or policy makers could easily understand the logic of the framework, facilitating informed decisions regarding EHR access control based on the insights of the framework.

Our framework demonstrated consistent response patterns across diverse scenarios, which substantially bolstered its interpretability. Provided satisfactory reasoning based on factors such as role-specific permissions, policy adherence, patient consent, healthcare purpose, and supervision. While processing requests and offering recommendations, it effectively accounted for various aspects defined by the My Health Records Act 2012. The analysis indicated a substantial degree of transparency and interpretability in the framework’s decision-making process, increasing its potential utility in a real-world healthcare setting. Although these promising results are encouraging, continued refinement and testing of the framework’s capabilities, particularly for complex scenarios, are necessary to further enhance its transparency and interpretability. Balancing this need with human oversight, especially in ambiguous or emotionally charged situations, is crucial. The transparency and interpretability assessment results of the GPT-Onto-CAABAC framework demonstrated its capacity to offer decision-making processes that are comprehensive, consistent, and accessible to end-users, thus suggesting its potential as a viable decision support tool in healthcare settings.

## Discussions

This section delves into a comprehensive discussion of the significant issues that emerged during the experiment.

### Challenges and overcoming strategies

The implementation of the GPT-Onto-CAABAC framework in healthcare, despite its significant potential, presents several salient challenges. The complexity of healthcare care scenario, performance and validity issues, and the overarching concern of societal trust necessitate a systematic address. However, these challenges also present opportunities for further refinement and innovation.

1. **Stability of GPT-generated texts**: In our pilot testing, we found that GPT produces slight variations in its outputs for the same input, primarily linguistic rather than semantic. We propose regular audits and ongoing scrutiny to ensure the consistency and reliability of GPT-generated content. Additionally, implementing feedback loops from end-users can provide valuable insights for model fine-tuning.
2. **Performance of the GPT models**: With the increasing sophistication and size of GPT models, there’s an associated increase in response generation time, making the framework unsuitable for real-time, time-critical decision-making in healthcare. To tackle this, we recommend continued performance evaluations and the development of optimization strategies. This may involve parallel processing, model pruning, or exploring hardware acceleration options.
3. **Validity of GPT-based decisions**: The potential of GPT models to produce hallucinations—factually incorrect or irrelevant outputs—could lead to non-compliant healthcare decisions [109]. To mitigate this risk, it is crucial to implement continuous validation checks and a verification mechanism. This might involve cross-checking GPT outputs with trusted resources, implementing peer-review mechanisms, or integrating GPT with rule-based systems for sanity checks.
4. **Societal trust in AI systems**: The potential for hallucinations and the opaque nature of AI algorithms present a significant challenge in fostering societal trust. For this, we advocate for strong human oversight, robust mechanisms for GPT output validity monitoring, and effective public communication strategies. Transparency about model limitations, clear communication about how decisions are made, and maintaining accountability are essential in earning public trust. Additionally, collaboration with regulatory bodies and ethicists to design guidelines and policy frameworks can contribute to societal trust.

Addressing these challenges is not a one-time activity but requires an ongoing cycle of refining and evaluating the GPT-Onto-CAABAC framework. Through continuous iteration, we can improve performance, validate results, improve transparency, and maintain effective public communication to harness the power of this framework in healthcare decision making.

### Applications in healthcare settings

Our GPT-Onto-CAABAC framework offers an adaptable solution to suit a variety of healthcare settings. Its flexibility facilitates its employment in healthcare decision-making domains, acting as either a proactive recommendation system or a reactive risk management tool. Traditional healthcare security consultations are plagued by challenges such as the intensive manual work required to audit intricate policies, unclear interpretations of regulations, and the rigidity to adapt to new policies. These issues, combined with often inadequate insight, could affect the effectiveness of consultations. The GPT-Onto-CAABAC framework confronts these challenges head on. LLMs automate auditing, drastically reducing manual involvement. The natural language skills of GPT models clarify complex healthcare contexts, and the continual learning feature of the framework keeps it aligned with changing regulations. This combined prowess offers healthcare professionals a reliable decision-making tool.

Activistically, our framework guides early decision-making stages, presenting policy-aligned alternatives for complex clinical situations. Here, GPT models comprehend detailed patient data, while ontology systems provide context-driven advice based on policy and regulatory interpretations. This cohesive method promotes complex decision making tailored to each case’s specifics. As a reactive mechanism, the GPT-Onto-CAABAC system reviews healthcare decisions after the fact, ensuring that they adhere to legal and organizational standards while spotlighting nonconformities. This retrospective review ensures consistent policy adherence, highlights training needs, and pinpoints policy areas that need more clarity. In addition, this framework has potential as an educational asset in healthcare training. Through the analysis of previous decisions, it can refine academic syllabi, shedding light on the intricate relationship between healthcare methods, policy mandates, and genuine patient situations. Despite its evident value, it remains essential to evaluate the effectiveness of the GPT-Onto-CAABAC framework in diverse healthcare settings, ensuring its continued relevance and contribution to healthcare decision processes.

### Integrating human oversight in the GPT-Onto-CAABAC framework

The study addresses the Human Oversigh within the GPT-Onto-CAABAC framework, and we emphasize its critical role in enhancing the decision-making process. Although GPT and the CAABAC model offer robust automated capabilities for access control and conflict resolution, human oversight serves as an essential layer to ensure ethical compliance, accountability, and adaptability to complex scenarios that automated systems may not fully grasp. This integration allows for a comprehensive review of automated decisions, particularly in sensitive cases, ensuring that they are in accordance with organizational policies, legal standards, and ethical considerations. Through this collaborative approach, the framework not only leverages the efficiency of automation but also retains the discerning judgment of human experts, effectively resolving the potential limitations of relying solely on automated processes [152–156].

### Expanded use cases beyond the EHR

Our GPT-Onto-CAABAC framework has broad applicability across diverse sectors that require complex and detailed access control decisions considering compliance, context, and attributes. Below are some potential use cases:

1. **Financial Services:** In the financial sector, access controls for sensitive customer data need to balance privacy regulations, individual access needs, and security priorities. The framework can aid in compliant access control by considering financial advisor attributes, customer consent context, and privacy laws.
2. **Defense Organizations:** For defense organizations, granting access to classified data requires strict adherence to security protocols and hierarchies. The framework can incorporate user roles, context like emergency situations, and classification levels to make informed yet flexible access decisions.
3. **Legal Services:** In legal services, client confidentiality is paramount while collaborating with experts across specializations. The framework can weigh attorney attributes, client permissions, and legal ethics codes to enable secure yet productive information sharing.
4. **Public Sector:** Government agencies manage vast sensitive citizen data subject to complex regulations. The framework can help navigate user clearances, data types, compliance needs, and transparency laws for responsible public data access.
5. **Research Institutions:** Academic research requires collaborations across domains while protecting participant privacy. The framework can balance researcher credentials, study protocols, ethics approvals and privacy laws to uphold rigorous access control standards.

### Translating concept to real-world implementation

**Fig 5:**
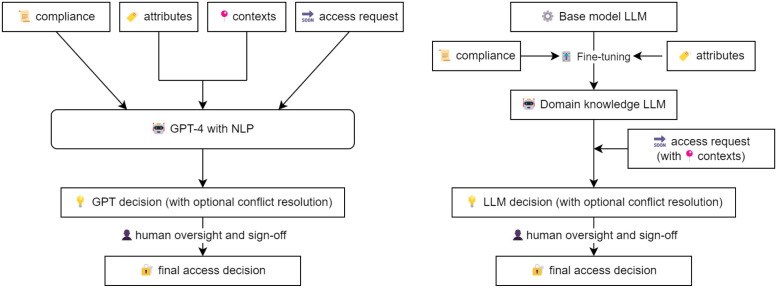
Comparison of our GPT-4-based prototype (left) and a practical domain knowledge LLM implementation (right).

While the GPT-Onto-CAABAC framework shows promise as a conceptual model, translating it into large-scale healthcare implementation requires the adoption of a fine-tuned domain knowledge LLM (Fig 5), and requires significant translational research and stakeholder participation. Some key aspects should be considered:

1. **Pilot Testing and Optimization**: Extensive testing across diverse healthcare contexts, institutions and geographic regions is crucial. This allows for framework optimization and customization based on lessons learned during deployment.
2. **Regulatory Approvals**: Securing approvals from healthcare governance bodies and demonstrating compliance is pivotal prior to full-scale rollout. This ensures patient safety and security standards are met.
3. **Change Management**: Training healthcare professionals on integrating the framework into workflows is vital. Managing organizational change and addressing adoption barriers smooths the transition.
4. **Patient Advocacy**: Incorporating patient perspectives through focus groups and consultation can identify potential ethical concerns early. Their insights further bolster framework transparency.
5. **Continuous Improvement**: Updating the framework as healthcare regulations and AI advance is imperative. Establishing processes for regular enhancements sustains long-term relevance.
6. **Economic Analysis**: Conducting cost-benefit analysis guides budgeting and resource allocation for development and maintenance. Quantifying value gained aids wider adoption.

Fig 5 illustrates a comparison between our GPT-4-based prototype (left) and a implementation of a large language model (LLM) of practical domain knowledge (LLM) (right). The figure highlights differences in performance, accuracy, and applicability between the two models, showcasing how our GPT-4-based approach leverages advanced natural language processing capabilities to provide more effective and contextually relevant responses compared to traditional domain-specific LLM implementations.

**Fig 6:**
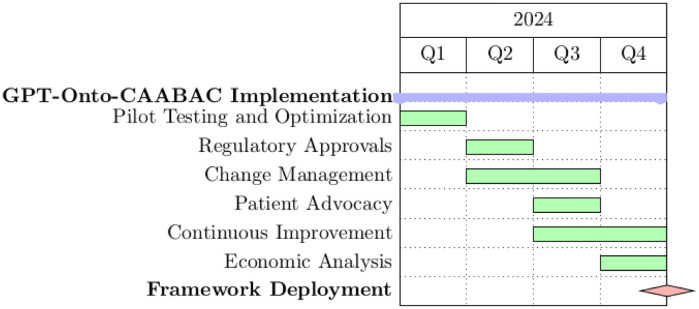
GPT-Onto-CAABAC implementation roadmap for 2024.

The Gantt chart, shown in Fig 6, visualizes the implementation timeline for 2024. The figure illustrates the GPT-Onto-CAABAC implementation roadmap, which outlines the planned stages and milestones to deploy the GPT-Onto-CAABAC model, including initial development phases, testing and validation, integration with existing systems, and full-scale deployment. The timeline provides a clear view of the project’s progress and key objectives to be achieved throughout the year. The chart has been derived based on expert estimates and stakeholder inputs:

1. **Pilot Testing and Optimization** is scheduled for Q1, considering it is the primary phase to validate the framework.
2. **Regulatory Approvals** are set in Q2, once preliminary results from pilot tests are available.
3. **Change Management** spans from Q2 to Q3, as training and transition management processes often overlap with other tasks.
4. **Patient Advocacy** is planned for Q3, ensuring ethical considerations are reviewed and integrated.
5. **Continuous Improvement** begins from Q3 and extends to Q4, emphasizing ongoing updates based on the framework’s deployment feedback.
6. **Economic Analysis** is conducted in Q4 to guide further resource allocation and budgeting decisions.

This phased translational approach is key to overcoming operational complexities and bridging the gap from the conceptual model to field deployment. With diligent pilot testing, stakeholder engagement, iterative improvements, and economic prudence, the GPT-Onto-CAABAC framework can progress from theory to practice.

### Comparative analysis and evaluation of GPT-Onto-CAABAC

The study extends the discussion to include a comparative analysis of the GPT-Onto-CAABAC framework against existing solutions. This analysis not only highlights our framework’s unique contributions, but also situates it within the broader landscape of AI and healthcare access control.

1. **Adaptability and Context-Awareness:** Unlike traditional access control systems, the GPT-Onto-CAABAC framework offers superior adaptability and context awareness, crucial for dynamic healthcare settings. Our framework’s use of GPT models and ontologies allows for a nuanced understanding of user roles and access needs in real-time, outperforming conventional systems that often require manual updates [55, 85, 155].
2. **Real-World Application and Scalability:** Through scenario-based evaluations, we demonstrate the practical applicability and scalability of the GPT-Onto-CAABAC framework. Compared to existing models, which are typically validated in controlled or small-scale settings, our approach is tested against a variety of complex healthcare scenarios, demonstrating its readiness for broader implementation [18, 161–163].
3. **Interpretability and Transparency:** The framework improves on the opacity often associated with AI systems. By integrating ontological reasoning with GPT’s natural language processing capabilities, it offers interpretability and transparency in decision-making, a step forward from the “black box” nature of many AI tools [40, 46].
4. **Security and Privacy:** Security and privacy considerations are paramount in our framework. Using contextually enriched attribute-based access control, GPT-Onto-CAABAC ensures that access decisions meet the highest standards of data protection, compared favorably with existing solutions that may not offer the same level of granular control [95, 113, 164].
5. **Integration with Existing Systems:** Our framework is designed for compatibility with existing EHR systems, facilitating seamless integration. This aspect is particularly noteworthy compared to other proposals that may require substantial modifications to current infrastructures [56, 82, 150, 165, 166].
6. **Future-Proofing and Flexibility:** Finally, the GPT-Onto-CAABAC framework is built with future developments in mind. Its modular design allows for easy updates as AI technology and healthcare practices evolve, offering a flexible solution that remains relevant over time [95, 161, 167, 168].

This comparative analysis underscores the innovative contributions of the GPT-Onto-CAABAC framework and its potential to address current and future challenges in healthcare access control.

### Research challenges and limitations

Our research presented in the article focuses primarily on the application of GPT models, ontology systems, and CAABAC models in the context of access control to the EHR. Some potential limitations of our research could include the following.

1. Research may be limited by the quality and quantity of data used to train GPT models. If the data are not diverse or comprehensive enough, the models might not perform optimally in real-world scenarios.
2. Research may also be limited by the complexity of integrating multiple systems (GPT models, ontology systems, and access control models). This integration might present challenges in terms of system compatibility, data synchronization, and performance optimization.
3. Research may be limited by the rapidly evolving nature of both healthcare regulations and AI technologies. The proposed framework might need to be continuously updated to keep up with these changes.

Although the GPT-Onto-CAABAC model offers significant advances in access control, it is essential to recognize the potential challenges and limitations associated with its implementation. These include the interpretability of AI-driven decisions, scalability issues, and resource requirements.

#### Interpretability of AI-Driven decisions

One of the main challenges with AI-driven models is the interpretability of decisions. The complex nature of GPT and ontology-based decision-making can make it difficult for stakeholders to understand the rationale behind specific access control decisions. To address this, the model incorporates explainability features that provide clear, human-readable justifications for each decision. However, ensuring that these explanations are consistently understandable between diverse user groups remains a challenge.

#### Scalability issues

Scalability is a critical concern when implementing a complex model such as GPT-Onto-CAABAC, particularly in large-scale healthcare environments with vast amounts of data and numerous access requests. The performance of the model can be affected by the computational overhead required to process real-time data and generate decisions dynamically. To mitigate this, the implementation must take advantage of efficient algorithms, distributed computing techniques, and robust infrastructure to handle large volumes of requests without significant latency.

#### Resource requirements

The resource requirements for training, deploying and maintaining the GPT-Onto-CAABAC model are substantial. These include the need for high-performance computing resources, extensive storage for large datasets, and continuous updates to the model based on new data and evolving regulations. Organizations must be prepared to invest in the necessary infrastructure and expertise to support the model’s operation. In addition, the energy consumption associated with running such a sophisticated model is a consideration for sustainable implementation.

#### Data privacy and security

While the model is designed to enhance security, it also presents potential risks related to data privacy. The integration of diverse data sources and the need for real-time processing could expose sensitive information if not managed correctly. Robust security measures, including encryption, access controls, and regular audits, are essential to protect against data breaches and ensure compliance with privacy regulations.

#### Continuous learning and adaptation

The dynamic nature of the healthcare environment and regulatory landscape requires continuous learning and adaptation of the model. This ongoing process requires regular updates and retraining of the model to maintain relevance and effectiveness. The challenge lies in ensuring that these updates are seamlessly integrated without disrupting the overall system’s functionality.

By recognizing these challenges and limitations, we can better prepare for the practical implementation of the GPT-Onto-CAABAC model, ensuring that it delivers on its promise of enhanced access control while addressing potential risks and constraints.

### Future research directions

Given the potential limitations of our study, we believe future research could focus on the following:

1. Improving the quality and diversity of the training data for the GPT models. This could involve collecting more data from a wider range of sources or developing new data augmentation techniques.
2. Converting the framework into a domain knowledge LLM tailored for specific use cases, as detailed in Section: Expanded use cases beyond the EHR.
3. Exploring more efficient ways to integrate GPT models, ontology systems, and access control models. This could involve developing new algorithms or system architectures.
4. Keep up to date with the latest developments in healthcare regulatory and AI technologies. This could involve regular literature reviews or collaborations with regulatory bodies and AI research institutions.

## Conclusion

Our proposed GPT-Onto-CAABAC framework represents a significant advancement in EHR access control by incorporating advanced AI capabilities, presenting a dynamic context-aware model. This integration has the potential to revolutionize healthcare data security and address the multifaceted complexities of EHR access control comprehensively. The ontology-driven component provides a structured methodology for defining crucial concepts such as users, resources, roles, permissions, and contextual data, underpinning coherent access policy articulation, thereby strengthening EHR security. The adaptability of the system increases through the integration of CAAC and ABAC, enhancing its applicability in various healthcare settings. With the inclusion of the GPT model, the system can take advantage of sophisticated NLP capabilities, facilitating the extraction and interpretation of complex legal and regulatory data, thus enriching decision-making processes. The design of our model promotes adaptability and efficiency while upholding accountability principles, with built-in mechanisms for human evaluation and oversight to foster responsible AI use.

Despite the promising results, it is essential to acknowledge the disadvantages and limitations observed during the evaluation.

1. **Complexity and Interpretability:** The model’s complexity can make it challenging for stakeholders to interpret the decision-making process. Although explainability features are integrated, ensuring consistent understanding across diverse user groups remains a challenge.
2. **Scalability Issues:** The computational overhead required for real-time context analysis and decision making can affect the model’s scalability in large-scale healthcare environments. Optimizing performance to handle high volumes of access requests without latency is an area that needs further enhancement.
3. **Resource Requirements:** The model requires substantial computational resources to train, deploy, and maintain. High-performance computing infrastructure and continuous updates are necessary, which can be resource-intensive.
4. **Data Privacy Concerns:** Integrating diverse data sources and real-time processing could expose sensitive information if not managed correctly. Robust security measures are essential to mitigate the risks associated with data breaches.

Despite these disadvantages, the model shows promising results in improving the flexibility and precision of access control decisions, as demonstrated by its performance in various healthcare roles. The dynamism, adaptability, robustness and context-sensitive attributes of the model enable it to meet evolving healthcare demands while adhering to the prevailing regulations and policies.

Future research will focus on addressing the identified limitations and enhancing the model’s capabilities:

1. **Improving Interpretability:** Developing advanced explainability tools to ensure that stakeholders can easily understand the decision-making process. This includes creating more user-friendly interfaces and detailed decision logs.
2. **Enhancing Scalability:** Implementing optimization techniques and exploring distributed computing approaches to efficiently handle large-scale data and high volumes of access requests.
3. **Reducing Resource Requirements:** Investigating more efficient algorithms and take advantage of cloud-based solutions to reduce computational resources needed for model training and deployment.
4. **Strengthening Data Privacy:** Enhancing security measures, including encryption and anonymization techniques, to protect sensitive data during integration and real-time processing.
5. **Extending Application Domains:** Exploring the applicability of the GPT-Onto-CAABAC model in other domains beyond healthcare, such as the finance and legal industries, to evaluate its versatility and robustness.

Beyond its immediate application in healthcare care, the proposed model shows considerable promise for broader implications. The inherent design of the model showcases immense potential for auditing access control decisions not only in healthcare, but across various sectors. Industries with multidimensional policies, rapidly changing contexts, and the need for detailed post-decision audits could significantly benefit from such a model. This opens avenues for the GPT-Onto-CAABAC framework to elevate access control auditing across many critical and dynamic environments.

In summary, while the GPT-Onto-CAABAC framework introduces significant advances in EHR access control, its reliance on advanced AI and ontology models introduces complexities in implementation and requires substantial computational resources. Future research could explore optimizing these aspects to enhance scalability and reduce overhead. Furthermore, the evolving nature of GPT models requires continuous monitoring for ethical and privacy implications, suggesting further refinement in incorporating robust ethical guidelines and privacy-preserving mechanisms. As the field progresses, we anticipate that the GPT-Onto-CAABAC model will continue to be a novel and adaptable solution, improving its efficacy in diverse healthcare scenarios, and pushing the limits of AI application in healthcare.

## Supporting information

S1 File Questions to evaluate reasoning on raw facts. (PDF)

## Glossary

ABAC: Attribute-Based Access Control
AI: Artificial Intelligence
CAABAC: Context-Aware Attribute-Based Access Control
CAAC: Context-Aware Access Control
EHR: Electronic Health Record
GPT: Generative Pre-trained Transformers
LLM: Large Language Model
NLP: Natural Language Processing
RBAC: Role-Based Access Control
