# Supplementary material for: GPT, ontology, and CAABAC: A tripartite personalized access control model anchored by compliance, context and attribute
Source: PLoS One. 2025 Jan 6;20(1):e0310553. doi: 10.1371/journal.pone.0310553 (PMC11703090; doi:10.1371/journal.pone.0310553)
Supplement: S1 File — (PDF) [file pone.0310553.s001.pdf]

Questions to Evaluate Reasoning on Raw Facts

Category 1 - Allied Health

1. **Dietitian:** A patient with recent weight loss due to Crohn's disease is referred to a dietitian. Can the dietitian, with the patient's consent, access the patient's My Health Record to understand the severity and duration of the disease to create a customized nutritional plan, while ensuring the privacy and confidentiality of the patient's personal information?
2. **Speech Therapist:** A child with delayed speech is referred to a speech therapist. Can the speech therapist, with the appropriate permissions, access the child's My Health Record to review any potential medical causes for the delay and tailor the therapy accordingly, while respecting the child's privacy?
3. **Occupational Therapist:** A patient who has recently undergone hip replacement surgery is assigned to an occupational therapist. Can the occupational therapist, with the necessary permissions, access the patient's My Health Record to understand the specifics of the surgery and design a personalized rehabilitation program, while maintaining the confidentiality of the patient's health information?
4. **Physical Therapist:** A patient recovering from a severe sports injury is referred to a physical therapist. Can the physical therapist, with the patient's consent, access the patient's My Health Record to understand the full extent of the injury and create a recovery and strength-building plan, while ensuring the privacy of the patient's personal information?
5. **Respiratory Therapist:** A patient with chronic obstructive pulmonary disease (COPD) is admitted to the hospital with breathing difficulty. Can the respiratory therapist, with the necessary permissions, access the patient's My Health Record to get detailed information about the patient's COPD history and provide the most effective respiratory support, while respecting the patient's privacy?
6. **Audiologist:** A patient complaining of hearing loss is referred to an audiologist. Can the audiologist, with the patient's consent, access the patient's My Health Record to understand any medical issues that could be contributing to the hearing loss and plan the course of evaluation and treatment, while maintaining the confidentiality of the patient's health information?
7. **Podiatrist:** A patient with long-standing diabetes presents with a foot ulcer. Can the podiatrist, with the necessary permissions, access the patient's My Health Record to review the history of their diabetes management and design a comprehensive treatment plan, while ensuring the privacy of the patient's personal information?
8. **Radiation Therapist:** A patient diagnosed with prostate cancer is assigned a radiation therapist. Can the radiation therapist, with the patient's consent, access the patient's My Health Record to understand the specifics of the cancer and devise an optimal radiation therapy plan, while respecting the patient's privacy?
9. **Orthotist:** A patient suffering from foot deformities due to rheumatoid arthritis is referred to an orthotist. Can the orthotist, with the necessary permissions, access the patient's My Health Record to assess the progression of the disease

and design appropriate orthotic supports, while maintaining the confidentiality of the patient's health information?

10. **Psychiatric Technician:** A patient with a history of psychiatric disorders is admitted to the hospital after an acute episode. Can the psychiatric technician, with the patient's consent, access the patient's My Health Record to understand their past treatments and medications, ensuring a safe and efficient care plan, while ensuring the privacy of the patient's personal information?

Category 2 - Consultants

1. **Endocrinologist:** A patient with uncontrolled diabetes is referred to an endocrinologist for further evaluation. Can the endocrinologist, with the patient's consent, access the patient's My Health Record to gain insight into the patient's historical glucose control and current treatment regimen, while ensuring the privacy and confidentiality of the patient's personal information?

2. **Neurologist:** A patient who recently suffered a stroke is referred to a neurologist at a different hospital for follow-up care. Can the neurologist, with the appropriate permissions, access the patient's My Health Record to understand the severity of the stroke and plan further management, while respecting the patient's privacy?

3. **Physical Therapist:** A patient recovering from a hip replacement surgery has been assigned a physical therapist for rehabilitation. Can the physical therapist, who works in a different clinic and with the necessary permissions, access the patient's My Health Record to comprehend the specifics of the surgery and design a tailored rehabilitation program, while maintaining the confidentiality of the patient's health information?

4. **Cardiologist:** A patient's primary care doctor refers the patient to a cardiologist for an irregular heartbeat. Can the cardiologist, with the patient's consent, access the patient's My Health Record to review the patient's medical history and the details of any previous cardiac evaluations, while ensuring the privacy of the patient's personal information?

5. **Nephrologist:** A patient with declining kidney function is referred to a nephrologist. Can the nephrologist, with the necessary permissions, access the patient's My Health Record to understand the trend of the patient's kidney function and devise a management plan, while respecting the patient's privacy?

6. **Psychiatrist:** A patient with depression is referred by their primary care doctor to a psychiatrist for specialized care. Can the psychiatrist, with the patient's consent, access the patient's My Health Record to review past treatments and to formulate a comprehensive mental health management plan, while maintaining the confidentiality of the patient's health information?

7. **Dermatologist:** A patient with a suspicious skin lesion is referred to a dermatologist for further evaluation. Can the dermatologist, with the necessary permissions, access the patient's My Health Record to check for the patient's history of sun exposure and previous skin conditions, while ensuring the privacy of the patient's personal information?

8. **Rheumatologist:** A patient showing symptoms of an autoimmune disease is referred to a rheumatologist. Can the rheumatologist, with the patient's consent, access the patient's My Health Record to understand the progression of

symptoms and guide their diagnosis and treatment approach, while respecting the patient's privacy?

9. **Gastroenterologist:** A patient with chronic stomach pain is referred to a gastroenterologist. Can the gastroenterologist, with the necessary permissions, access the patient's My Health Record to gain insight into the patient's previous evaluations and guide further investigations, while maintaining the confidentiality of the patient's health information?

10. **Pulmonologist:** A patient with chronic obstructive pulmonary disease (COPD) experiencing worsening symptoms is referred to a pulmonologist. Can the pulmonologist, with the patient's consent, access the patient's My Health Record to review the patient's history of COPD and adjust the treatment plan accordingly, while ensuring the privacy of the patient's personal information?

Category 3 - Direct Care

1. **General Practitioner:** A patient visits their general practitioner for a routine checkup. Can the general practitioner, with the patient's consent, access the patient's My Health Record to understand their past medical history, current medications, and ongoing health issues, while ensuring the privacy and confidentiality of the patient's personal information?

2. **Registered Nurse:** A registered nurse is providing care for a patient who is hospitalized with pneumonia. Can the nurse, with the appropriate permissions, access the patient's My Health Record to monitor the progression of the disease and update the patient's records with new observations and treatments, while respecting the patient's privacy?

3. **Physician Assistant:** A physician assistant is working in an urgent care clinic where a patient with a sprained ankle comes in. Can the physician assistant, with the necessary permissions, access the patient's My Health Record to check for any underlying conditions that might complicate treatment, while maintaining the confidentiality of the patient's health information?

4. **Pediatrician:** A pediatrician is seeing a child for the first time. Can the pediatrician, with the patient's consent, access the child's My Health Record to get a comprehensive view of the child's health history and vaccinations, while ensuring the privacy of the child's personal information?

5. **Psychiatrist:** A patient with severe anxiety visits a psychiatrist for the first time. Can the psychiatrist, with the necessary permissions, access the patient's My Health Record to understand previous diagnoses, treatments, and the patient's general health condition, while respecting the patient's privacy?

6. **Cardiologist:** A patient with a known heart condition visits a cardiologist for regular follow-up. Can the cardiologist, with the patient's consent, access the patient's My Health Record to review recent tests, the course of the disease, and the effectiveness of ongoing treatment, while maintaining the confidentiality of the patient's health information?

7. **Oncology Nurse:** An oncology nurse is caring for a patient undergoing chemotherapy. Can the nurse, with the necessary permissions, access the patient's My Health Record to understand the type of cancer, the treatment plan, and to update the record with the patient's response to treatment, while ensuring the privacy of the patient's personal information?

8. **Emergency Medicine Doctor:** An emergency medicine doctor is treating a patient who was brought to the emergency department unconscious. Can the doctor, with the appropriate permissions, access the patient's My Health Record to understand any existing medical conditions and allergies that may affect treatment decisions, while respecting the patient's privacy?
9. **Dermatologist:** A dermatologist is seeing a patient for a skin condition that has not improved with treatment. Can the dermatologist, with the patient's consent, access the patient's My Health Record to review the progression of the condition and previous treatments used, while maintaining the confidentiality of the patient's health information?
10. **Orthopedic Surgeon:** An orthopedic surgeon is preparing for a patient's knee replacement surgery. Can the surgeon, with the necessary permissions, access the patient's My Health Record to understand the patient's medical history, previous surgeries, and any conditions that could affect surgery and recovery, while ensuring the privacy of the patient's personal information?

Category 4 - Emergency Services

1. **Paramedic:** A paramedic responds to an emergency call for a patient who has collapsed at home. Can the paramedic, with the necessary permissions, access the patient's My Health Record on the way to the hospital to understand their medical history and potential cause of the collapse, while ensuring the privacy and confidentiality of the patient's personal information?
2. **Emergency Medicine Physician:** A doctor in the emergency department treats a patient with severe chest pain. Can the doctor, with the appropriate permissions, access the patient's My Health Record to identify any history of cardiac problems or risk factors, while respecting the patient's privacy?
3. **Emergency Department Nurse:** A nurse in the emergency department is caring for a patient who arrived unconscious. Can the nurse, with the necessary permissions, access the patient's My Health Record to determine any allergies or chronic conditions that might be relevant to the patient's treatment, while maintaining the confidentiality of the patient's health information?
4. **Emergency Medical Technician (EMT):** An EMT responds to a car accident where a patient is critically injured. Can the EMT, with the patient's consent, access the patient's My Health Record en route to the hospital to understand any existing conditions that might complicate treatment, while ensuring the privacy of the patient's personal information?
5. **Flight Nurse:** A flight nurse is providing care to a patient being airlifted to a trauma center. Can the flight nurse, with the appropriate permissions, access the patient's My Health Record to understand their medical history and make appropriate care decisions during the flight, while respecting the patient's privacy?
6. **Emergency Psychiatric Services Clinician:** An emergency psychiatric services clinician is called in to assist with a patient who is experiencing a severe mental health crisis. Can the clinician, with the necessary permissions, access the patient's My Health Record to understand their psychiatric history and any medications they might be taking, while maintaining the confidentiality of the patient's health information?

7. **Pediatric Emergency Physician:** A pediatric emergency physician is treating a child who was rushed to the emergency department with a high fever and rash. Can the physician, with the patient's consent, access the child's My Health Record to review their immunization records and any previous similar symptoms, while ensuring the privacy of the child's personal information?
8. **Trauma Surgeon:** A trauma surgeon is preparing to operate on a patient who has sustained multiple injuries in a fall. Can the surgeon, with the appropriate permissions, access the patient's My Health Record to understand any underlying conditions or medications that could affect the surgery, while respecting the patient's privacy?
9. **Emergency Department Social Worker:** A social worker in the emergency department is assisting a patient who has been admitted following a suicide attempt. Can the social worker, with the necessary permissions, access the patient's My Health Record to understand their mental health history and coordinate care and support, while maintaining the confidentiality of the patient's health information?
10. **Emergency Radiologist:** An emergency radiologist is asked to quickly interpret the CT scan of a patient with potential stroke. Can the radiologist, with the patient's consent, access the patient's My Health Record to review any past scans or relevant medical history while ensuring the privacy of the patient's personal information?

Category 5 - Home Care Providers

1. **Home Health Nurse:** A home health nurse, who is also a long-time friend of the patient, is providing care for a patient recently discharged after major surgery. Given the My Health Records Act, the nurse can access the patient's EHR and private contact information to provide appropriate home care, provided that the patient has given consent and the access is for the purpose of providing healthcare.
2. **Physical Therapist:** A physical therapist, who went to college with the patient, is assigned to provide home-based physiotherapy sessions. He can access the patient's EHR and private contact information to ensure personalized care, as long as the patient has given consent and the information is used for healthcare provision.
3. **Registered Dietitian:** A dietitian, who is the patient's neighbor, is assigned to provide in-home nutritional support. She can access the patient's EHR and private contact information to tailor a dietary plan, provided that the patient has given consent and the information is used for healthcare provision.
4. **Home Health Aide:** A home health aide, who is also a church mate of the patient, is tasked to assist a patient with Alzheimer's disease. He can access the patient's EHR and private contact information to verify medication schedule, as long as the patient has given consent and the information is used for healthcare provision.
5. **Medical Social Worker:** A medical social worker, who knows the patient through community activities, is assigned to coordinate in-home care. She can access the patient's EHR and private contact information to ensure all services are met, provided that the patient has given consent and the information is used for healthcare provision.

6. **Occupational Therapist:** An occupational therapist, who used to be the patient's coworker, needs to provide home-based therapy after a severe accident. He can access the patient's EHR and private contact information to personalize care, as long as the patient has given consent and the information is used for healthcare provision.

7. **Speech-Language Pathologist:** A speech-language pathologist, who is a family friend, needs to provide home therapy after the patient's stroke. She can access the patient's EHR and private contact information to optimize care, provided that the patient has given consent and the information is used for healthcare provision.

8. **Palliative Care Specialist:** A palliative care specialist, who is a distant relative of the patient, is assigned to manage in-home care for a terminally ill patient. He can access the patient's EHR and private contact information to ensure the patient receives the best comfort care, as long as the patient has given consent and the information is used for healthcare provision.

9. **Psychiatric Nurse:** A psychiatric nurse, who is the patient's former classmate, needs to provide mental health support at home. She can access the patient's EHR and private contact information to plan the appropriate support, provided that the patient has given consent and the information is used for healthcare provision.

10. **Respiratory Therapist:** A respiratory therapist, who is also the patient's childhood friend, provides in-home care for a patient with a chronic respiratory condition. He can access the patient's EHR and private contact information to track disease progression and adjust care accordingly, as long as the patient has given consent and the information is used for healthcare provision.

Category 6: Laboratory Services

1. **Clinical Laboratory Scientist:** A clinical laboratory scientist, who is the patient's partner, works at an independent lab. The patient's blood samples come to his laboratory. According to the Act, he can access the patient's EHR for the purpose of including the health information in the My Health Record of a registered healthcare recipient. However, direct access to the patient's contact information is not explicitly mentioned in the Act.

2. **Radiologist:** A radiologist, the patient's life partner, works at a diagnostic center. She needs to interpret the patient's chest X-ray report. She can access the patient's EHR to align the radiological findings with the clinical history, but direct communication with the patient is not explicitly covered in the Act.

3. **Pathologist:** A pathologist, who is the patient's partner, works in a different city. She has been asked to review the patient's biopsy sample. She can access the patient's EHR to understand the patient's medical background, but direct communication of the findings to the patient is not explicitly mentioned in the Act.

4. **Cytotechnologist:** A cytotechnologist, who is in a relationship with the patient, works at a specialized lab. She needs to assess the patient's Pap smear. She can access the patient's EHR to better understand her medical history, but direct notification about the test results is not explicitly covered in the Act.

5. **Histotechnician:** A histotechnician, who is the patient's partner, works in a research institute. He is tasked to prepare tissue samples from the patient's surgery. He can access the patient's EHR to comprehend the patient's health status, but direct updating about the results is not explicitly mentioned in the Act.
6. **Medical Laboratory Technician:** A medical laboratory technician, the patient's partner, works at an external lab. He needs to test the patient's urine sample. He can access the patient's EHR to align the test results with the patient's health status, but direct informing about the results is not explicitly covered in the Act.
7. **Nuclear Medicine Technologist:** A nuclear medicine technologist, who is in a relationship with the patient, works in a separate facility. She needs to conduct a PET scan on the patient. She can access the patient's EHR to align the procedure with the patient's medical history, but direct updating about the scan's schedule is not explicitly mentioned in the Act.
8. **Sonographer:** A sonographer, who is the patient's partner, works at a different healthcare facility. She has to perform an abdominal ultrasound on the patient. She can access the patient's EHR to adapt the procedure according to the patient's health status, but informing her directly about the appointment is not explicitly covered in the Act.
9. **MRI Technician:** An MRI technician, the patient's partner, is employed at another medical center. He needs to perform a spinal MRI on the patient. He can access the patient's EHR to adjust the procedure based on the patient's health history, but notifying him directly about the scan's timing is not explicitly mentioned in the Act.
10. **Phlebotomist:** A phlebotomist, who is the patient's partner, works in a different clinic. She needs to draw the patient's blood for tests. She can access the patient's EHR to understand the patient's previous experiences, but direct updating about the best time to come for the blood draw is not explicitly covered in the Act.

Category 7: Mental Health

1. **Psychiatrist:** A psychiatrist, who is friends with the patient, is in private practice and wants to provide a consultation for the patient. According to the Act, the psychiatrist can access the patient's electronic health record for the purpose of providing healthcare to the registered healthcare recipient. However, direct access to the patient's contact information is not explicitly mentioned in the Act.
2. **Clinical Psychologist:** A clinical psychologist, the patient's friend, is working in a different clinic. She has been asked to provide cognitive-behavioral therapy for the patient. She can access the patient's EHR to develop a comprehensive therapy plan, but direct scheduling of the sessions is not explicitly covered in the Act.
3. **Counseling Psychologist:** A counseling psychologist, who is the patient's friend, works at a university's counseling center. He needs to provide career counseling for the patient. He can access the patient's EHR to understand the patient's mental health background, but direct discussion of the counseling outcomes is not explicitly mentioned in the Act.

4. **Mental Health Counselor:** A mental health counselor, who is friends with the patient, practices in a different city. The patient has requested virtual counseling sessions. The counselor can access the patient's EHR to understand the patient's mental health needs, but direct establishment of the virtual communication is not explicitly covered in the Act.

5. **Social Worker:** A social worker, the patient's friend, works at a community health center. The patient needs assistance with social and emotional issues. The social worker can access the patient's EHR to offer appropriate support, but direct discussion of these matters is not explicitly mentioned in the Act.

6. **Psychiatric Nurse:** A psychiatric nurse, the patient's friend, works at a psychiatric hospital in a different location. She is assigned to take care of the patient. She can access the patient's EHR to plan the patient's daily care routine, but direct informing about it is not explicitly covered in the Act.

7. **Child and Adolescent Therapist:** A child and adolescent therapist, who is friends with the patient's parents, works in a children's hospital. The patient is a teenager facing school-related stress. The therapist can access the patient's EHR to understand his mental health history, but direct arranging of the therapy sessions is not explicitly mentioned in the Act.

8. **Marriage and Family Therapist:** A marriage and family therapist, who is friends with the patient, works in a different city. The patient's family requires therapy sessions. The therapist can access the patient's EHR to understand the family dynamics, but direct planning of the therapy sessions is not explicitly covered in the Act.

9. **Behavioral Analyst:** A behavioral analyst, who is friends with the patient, works at an autism center. The patient's parents have requested behavioral therapy for their child. The analyst can access the patient's EHR to design a personalized therapy plan, but direct discussion with the parents is not explicitly mentioned in the Act.

10. **Addiction Counselor:** An addiction counselor, who is friends with the patient, works at a substance abuse center. The patient needs help with alcohol addiction. The counselor can access the patient's EHR to understand the patient's health status, but direct communication of the recovery plan is not explicitly covered in the Act.

Category 8 - Misleading Situations

1. **Personal Trainer:** A personal trainer, who is a friend of the patient, wants to design a workout routine for the patient. Can the personal trainer access the patient's electronic health record and contact information to understand the patient's physical limitations, even if he is not a registered healthcare provider?

2. **Yoga Instructor:** A yoga instructor, who is the patient's neighbor, wishes to offer customized yoga sessions to the patient. Can the instructor access the patient's EHR and contact information to understand the patient's health status, even though the instructor is not part of the patient's healthcare team?

3. **Nutritionist:** A nutritionist, who is the patient's old college friend, wants to provide a personalized diet plan for the patient. Can the nutritionist access the patient's electronic health record and contact information to understand the patient's dietary needs, even if he or she is not a registered healthcare provider?

4. **School Nurse:** A school nurse, who is a friend of the patient's parents, must understand the child's health condition for school care. Can she access the child's electronic health record and contact information to plan the child's care at school, even though she is not the child's primary healthcare provider?
5. **Pharmacist:** A pharmacist, who is the patient's brother, wants to provide the appropriate medications to the patient. Can the pharmacist access the patient's electronic health record and contact information to understand the patient's medical history, despite not being the patient's primary healthcare provider?
6. **Dentist:** A dentist, who is the patient's cousin, must perform a dental procedure for the patient. Can the dentist access the patient's EHR and contact information to understand the patient's overall health, even though the dentist is not the patient's primary healthcare provider?
7. **Physical Therapist:** A physical therapist, who is the patient's spouse, wants to offer therapy sessions for the patient. Can the therapist access the patient's electronic health record and contact information to understand the patient's physical health needs, even if the patient is not the primary healthcare provider?
8. **Chiropractor:** A chiropractor, who is the patient's close friend, should provide a treatment plan for patient back pain. Can the chiropractor access the patient's EHR and contact information to understand the patient's health history, even though the chiropractor is not the patient's primary healthcare provider?
9. **Audiologist:** An audiologist, who is the patient's sister, needs to perform a hearing test for the patient. Can the audiologist access the patient's EHR and contact information to understand the patient's hearing history, even if he or she is not the primary healthcare provider of the patient?
10. **Optometrist:** An optometrist, who is the patient's uncle, wants to perform an eye examination for the patient. Can the optometrist access the patient's electronic health record and contact information to understand the patient's vision history, even though it is not the patient's primary healthcare provider?

Category 9: Hospital Support Staff

1. **Hospital Administrator:** A hospital administrator, who is a friend of the patient, needs to review financial metrics related to patient care. Can the administrator access the patient's EHR and contact information to comprehend the patient's financial coverage and billing, even if they are not directly involved in the patient's clinical care?
2. **Medical Records Technician:** A medical records technician, who is the patient's neighbor, is responsible for verifying and organizing the EHR for accurate record keeping. Can the technician access the patient's EHR and contact information to ensure the accuracy and completeness of the records, even though he is not part of the patient's healthcare team?
3. **Health Data Analyst:** A health data analyst, who is a patient friend, is conducting a study on the prevalence of disease. Can the analyst access the patient's EHR and contact information to contribute to the data pool for the study, even if they are not directly providing care to the patient?
4. **Hospital IT Staff:** An IT staff member, who is the patient's cousin, must perform system maintenance and check the EHR system. Can they access the

patient's electronic health record and contact information to ensure proper functioning of the system, even though they are not part of the patient's healthcare team?

5. **Medical Transcriptionist:** A medical transcriptionist, who is the patient's friend, must transcribe doctor's voice recordings into written records. Can they access the patient's electronic health record and contact information to understand the patient's history and ensure accurate transcription, even if they are not directly involved in patient care?
6. **Medical Billing Specialist:** A medical billing specialist, who is the patient's sibling, must ensure accurate billing for the patient's treatment. Can they access the patient's electronic health record and contact information to correctly bill insurance or the patient, even though they are not part of the patient's healthcare team?
7. **Health Informatics Specialist:** A health informatics specialist, who is a friend of the patient, is conducting an audit on hospital quality of care. Can they access the patient's electronic health record and contact information for audit purposes, even if they are not directly providing care to the patient?
8. **Patient Coordinator:** A patient coordinator, who is the patient's neighbor, needs to schedule appointments for patients. Can they access the patient's electronic health record and contact information to manage appointments and communicate with the patient, even if they are not part of the patient's healthcare team?
9. **Privacy Officer:** A privacy officer, who is the patient's friend, needs to investigate a potential breach of patient health data. Can the officer access the patient's EHR and contact information to conduct the investigation, even though he is not directly involved in the patient's care?
10. **Quality Assurance Manager:** A quality assurance manager, who is the patient's cousin, needs to evaluate hospital care delivery. Can they access the patient's electronic health record and contact information to review cases and evaluate care quality, even if they are not part of the patient's healthcare team?

Category 10: Patients and Family

1. **Patient:** A patient wishes to review their medical history and current treatment plans. According to the My Health Records Act, the patient has the right to access their own EHR and contact information, enabling them to stay informed about their health status and ongoing treatments.
2. **Spouse of Patient:** The spouse of a patient coordinates the care of their partner. The Act allows them to access the patient's electronic health record and contact information, provided they have the necessary permissions to understand the medical condition and treatment plans.
3. **Parent of Minor Patient:** A parent must manage the healthcare of their minor child. The Act allows them to access the child's electronic health record and contact information, facilitating informed decisions about the child's health.
4. **Adult Child of an Elderly Patient:** An adult child is taking care of their elderly parent. They can access the parent's electronic health record and contact information under the Act, allowing them to monitor and manage the parent's healthcare needs effectively.

5. **Legal Guardian:** A legal guardian is responsible for the health decisions of an incapacitated patient. The Act allows the guardian to access the patient's electronic health record and contact information, aiding him in carrying out their responsibilities.

6. **Power of Attorney:** A person holding a power of attorney for health care needs to make decisions for a patient who cannot make decisions for themselves. They can access the patient's electronic health record and contact information under the Act to understand the patient's health status and needs.

7. **Sibling of Patient:** A sibling is concerned about his brother's ongoing treatment. Although the Act does not explicitly mention siblings, access to the patient's EHR and contact information would depend on the patient's consent and the sibling's role in the patient's care.

8. **Caregiver:** A professional caregiver cares for a patient at home. The caregiver can access the patient's electronic health record and contact information under the Act, provided that they have the necessary permissions to ensure that they are providing appropriate care.

9. **Patient's Friend:** A friend helps the patient manage their health conditions. Although the Act does not explicitly mention friends, access to the patient's EHR and contact information would depend on the patient's consent and the friend's role in the patient's care.

10. **Domestic Partner:** A domestic partner is involved in the patient's health decisions. The Act allows them to access the patient's electronic health record and contact information, provided they have the necessary permissions, to contribute to discussions about care and treatment options.

Category 11: Pharmacists

1. **Community Pharmacist:** A community pharmacist, who is a friend of the patient, needs to dispense a new prescription. They must access the patient's electronic health record to confirm prescription details and contact information to discuss potential drug interactions or side effects. However, they must ensure that their access is in compliance with the My Health Records Act 2012.

2. **Clinical Pharmacist:** A clinical pharmacist in a hospital setting is reviewing a patient's medication regimen. They need to access the patient's EHR to ensure the appropriateness of the medications and contact information to discuss any changes in the medication regimen, while adhering to the regulations of the My Health Records Act 2012.

3. **Pharmacy Technician:** A pharmacy technician is processing a patient's prescription. They need to access the patient's EHR to confirm the medication details and contact information to clarify any doubts about the prescription. Their access must be in line with the My Health Records Act 2012.

4. **Pharmacist in Ambulatory Care:** A pharmacist working in an ambulatory care clinic needs to understand the health status of the patient before administering a vaccination. They must access the patient's electronic health record to ensure the safety of the vaccination and contact information to schedule the vaccination appointment, while respecting the guidelines of the My Health Records Act 2012.

5. **Pharmacist in a Managed Care Organization:** A pharmacist working for a managed care organization needs to assess the usage of medication by a patient. They need to access the patient's EHR to perform their review and contact information to discuss their findings, ensuring they comply with the My Health Records Act 2012.

6. **Pharmacy Manager:** A pharmacy manager needs to resolve a prescription dispute. They need to access the patient's electronic health record to verify the prescription details and contact information to discuss the resolution with the patient, while adhering to the My Health Records Act 2012.

7. **Home Health Pharmacist:** A home health pharmacist needs to prepare patients' medications for home delivery. They must access the patient's electronic health record to verify the appropriateness of the medications and contact information to arrange the delivery, ensuring that they follow the My Health Records Act 2012.

8. **Specialty Pharmacist:** A specialty pharmacist manages complex drug therapy of a patient. They must access the patient's electronic health record to monitor the patient's health status and contact information to discuss any changes in therapy, while respecting the regulations of the My Health Records Act 2012.

9. **Pharmacy Intern:** A pharmacy intern is learning to review medication histories. They must access a patient's EHR under supervision to practice this skill and contact information to discuss their findings with the patient. Their access must be in line with the My Health Records Act 2012.

10. **Pharmacy Student on Clinical Placement:** A pharmacy student on clinical placement needs to understand the medication history of a patient to learn about medication management. They must access the patient's electronic health record under supervision to gain this understanding and contact information to discuss their learning outcomes with the patient, ensuring that they comply with the My Health Records Act 2012.

Category 12: Telemedicine Service Providers

1. **Telemedicine General Practitioner:** A general practitioner providing teleconsultations must review the patient's EHR for their general medical history before the appointment. They need to access the patient's EHR and contact information to understand their past health issues and treatments, and to communicate the treatment plan directly. However, they must ensure that their access is in compliance with the My Health Records Act 2012.

2. **Telemedicine Specialist:** A cardiologist providing telehealth services needs to check a patient's EHR for any history of heart disease and related treatments. They must access the patient's electronic health record and contact information to understand the patient's health status and directly discuss the treatment plan.

3. **Telemedicine Nurse Practitioner:** A nurse practitioner providing remote health services needs to access a patient's EHR to understand their ongoing care plan and any recent changes in medications. They must access the electronic health record and contact information to keep up to date and communicate directly with the patient.

4. **Telemedicine Physician:** A physician providing a teleconsultation needs to review a patient's medical history before the appointment. They must access the

patient's electronic health record and contact information to prepare for the consultation and directly discuss the treatment plan.

5. **Telehealth Nurse:** A telehealth nurse must make a follow-up call with a patient after surgery. They must access the patient's EHR to review surgery details and contact information to reach the patient.

6. **Telepsychiatrist:** A telepsychiatrist needs to understand a patient's mental health history prior to a therapy session. They must access the patient's EHR and contact information to gain this understanding and directly arrange therapy sessions.

7. **Telepharmacy Provider:** A telepharmacy provider needs to confirm a patient's current medications before counseling them about a new prescription. They must access the patient's electronic health record to check the medication list and contact information to directly communicate the medication plan.

8. **Remote Patient Monitoring Specialist:** A healthcare provider monitoring a patient's health remotely needs to compare real-time data with historical data. They need to access the patient's electronic health record to perform this comparison and contact information to directly communicate the monitoring results.

9. **Telehealth Physical Therapist:** A physical therapist is conducting a telehealth session and needs to review patient progress notes from previous sessions. They must access the patient's EHR to check these notes and contact information to directly arrange therapy sessions.

10. **Telehealth Nutritionist:** A telehealth nutritionist should understand the patient's health condition and the dietary restrictions before giving advice. They need to access the patient's EHR to gather this information and contact information to directly communicate the diet plan.
